# Master regulator analysis of paragangliomas carrying *SDHx*, *VHL*, or *MAML3* genetic alterations

**DOI:** 10.1186/s12885-019-5813-z

**Published:** 2019-06-24

**Authors:** John A. Smestad, L. James Maher

**Affiliations:** 1 Mayo Clinic Medical Scientist Training Program, Mayo Clinic College of Medicine and Science, Rochester, MN USA; 2 Department of Biochemistry and Molecular Biology, Mayo Clinic College of Medicine and Science, Rochester, MN USA

**Keywords:** Paraganglioma, Pheochromocytoma, Succinate dehydrogenase, von Hippel-Lindau, Mastermind-like transcriptional coactivator 3, Transcriptional network, Transcription factor, Retinoic acid

## Abstract

**Background:**

Succinate dehydrogenase (SDH) loss and mastermind-like 3 (*MAML3*) translocation are two clinically important genetic alterations that correlate with increased rates of metastasis in subtypes of human paraganglioma and pheochromocytoma (PPGL) neuroendocrine tumors. Although hypotheses propose that succinate accumulation after SDH loss poisons dioxygenases and activates pseudohypoxia and epigenomic hypermethylation, it remains unclear whether these mechanisms account for oncogenic transcriptional patterns. Additionally, *MAML3* translocation has recently been identified as a genetic alteration in PPGL, but is poorly understood. We hypothesize that a key to understanding tumorigenesis driven by these genetic alterations is identification of the transcription factors responsible for the observed oncogenic transcriptional changes.

**Methods:**

We leverage publicly-available human tumor gene expression profiling experiments (*N* = 179) to reconstruct a PPGL tumor-specific transcriptional network. We subsequently use the inferred transcriptional network to perform master regulator analyses nominating transcription factors predicted to control oncogenic transcription in specific PPGL molecular subtypes. Results are validated by analysis of an independent collection of PPGL tumor specimens (*N* = 188). We then perform a similar master regulator analysis in SDH-loss mouse embryonic fibroblasts (MEFs) to infer aspects of SDH loss master regulator response conserved across species and tissue types.

**Results:**

A small number of master regulator transcription factors are predicted to drive the observed subtype-specific gene expression patterns in SDH loss and *MAML3* translocation-positive PPGL. Interestingly, although EPAS1 perturbation is detectible in SDH-loss and VHL-loss tumors, it is by no means the most potent factor driving observed patterns of transcriptional dysregulation. Analysis of conserved SDH-loss master regulators in human tumors and MEFs implicated ZNF423, a known modulator of retinoic acid response in neuroblastoma. Subsequent functional analysis revealed a blunted cell death response to retinoic acid in SDH-loss MEFs and blunted differentiation response in SDH-inhibited SH-SY5Y neuroblastoma cells.

**Conclusions:**

The unbiased analyses presented here nominate specific transcription factors that are likely drivers of oncogenic transcription in PPGL tumors. This information has the potential to be exploited for targeted therapy. Additionally, the observation that SDH loss or inhibition results in blunted retinoic acid response suggests a potential developmental etiology for this tumor subtype.

**Electronic supplementary material:**

The online version of this article (10.1186/s12885-019-5813-z) contains supplementary material, which is available to authorized users.

## Background

Pheochromocytoma and paraganglioma (PPGL) are rare, closely-related neuroendocrine tumors arising from the adrenal medulla and autonomic ganglia of the peripheral nervous system, respectively. Over the last two decades more than 20 potentially causative genetic alterations have been elucidated for PPGL, including mutations in genes involved in kinase signalling, hypoxic response, and tricarboxylic acid (TCA) cycle metabolism [1–25]. Although targeted therapies for specific PPGL genetic subtypes are not currently available, tumor mutation and genetic subtype information are clinically useful for determining patient prognosis. In particular, patients with mutations in genes encoding components of the succinate dehydrogenase (SDH) complex of the TCA cycle, tend toward shorter metastasis-free survival and shorter overall survival [16, 26].

Recently, a large-scale integrative genomic analysis of PPGL through The Cancer Genome Atlas (TCGA) Project described another important genetic alteration in PPGL: a translocation involving the *MAML3* gene. This translocation also correlates with poor patient prognosis and higher rates of metastasis [27]. Collectively, this study estimated that ~ 11% of PPGL patients carry germline mutations in the four genes (*SDHA*, *SDHB*, *SDHC*, *SDHD; SDHx*) encoding SDH subunits, and ~ 5% of PPGL patients have tumor DNA carrying the novel *MAML3* translocation. Most PPGL metastases arise in patients corresponding to one of these two genetic subtypes, making the deconvolution of their underlying oncogenic mechanisms an important clinical priority.

It remains unknown how SDH loss or *MAML3* translocation actually drives malignancy. Regarding the newly-described *MAML3* translocation, it is hypothesized that this translocation is somehow associated with Wnt pathway activation and DNA hypomethylation [27], but the mechanism is unknown. Regarding SDH loss, the current tumorigenesis hypothesis proposes bi-allelic loss of any of the *SDHx* genes, followed by competitive inhibition of dioxygenase enzymes by accumulated succinate. The result is constitutive activation of hypoxic signalling (“pseudohypoxia” by succinate inhibition of the prolyl hydroxylases normally responsible for HIF1A hydroxylation) and global hypermethylation of histones and DNA [28, 29]. We recently reviewed this mechanistic paradigm [30]. However, the respective tumorigenic roles of chronic hypoxic signalling and chromatin hypermethylation are unknown. Constitutive activation of hypoxic signalling is believed to occur in both SDH-loss and VHL-loss PPGL tumors. In the latter case, hypoxic signalling is thought to be constitutively activated due to a defect in *VHL*, the E3 ubiquitin ligase responsible for ubiquitination of hydroxylated HIF-alpha subunits, normally targeting them for proteasomal degradation. To date, however, no unbiased analysis has been performed to evaluate the extent to which HIF activation accounts for oncogenic transcription observed in SDH-loss and VHL-loss PPGL tumors. Additionally, it is not known whether other oncogenic transcriptional programs are cued in these tumors by dysregulated transcription factors.

We are ultimately interested in understanding the mechanistic linkage between specific gene defects and PPGL tumorigenesis. In the present work, we have therefore undertaken an unbiased master regulator analysis (MRA) to infer perturbed transcription factors that potentially explain the observed patterns of transcriptional dysregulation specific to SDH-loss, VHL-loss, and *MAML3* translocation PPGL subtypes. This strategy leverages the ARACNE information theoretical approach to generate robust inferred transcriptional networks from large numbers of PPGL tumor specimen gene expression profiling experiments reported in the TCGA-PCPG cohort (*N* = 179 specimens) [31]. Using the resultant inferred transcriptional networks, we perform MRA to infer transcription factors whose regulons (i.e. sets of downstream target genes) are differentially-expressed in a given PPGL subtype. This approach to transcriptional network inference (TNI) and MRA has previously been applied to successfully identify transcription factors responsible for tumorigenic gene expression patterns in other cancers [32–40].

In the current work, we perform MRA for SDH-loss, VHL-loss, and *MAML3* translocation PPGL sub-types, and infer a small number of MRs inferred to collectively control the majority of differentially-expressed genes for each subtype. We demonstrate a considerable MR overlap for SDH-loss and *VHL*-loss PPGL sub-types, with each sub-type also characterized by a unique set of MRs. Interestingly and surprisingly, HIF transcription factors are not found to be among the most potent MRs in SDH-loss PPGL, although activity-based assessment reveals some detectible increase in EPAS1 activity relative to tumors lacking SDH or VHL defects. Importantly, we also report that over 20% of the differentially-expressed genes in *MAML3* translocation PPGL are inferred to be downstream targets of transcription factor IRX4, encoded by a gene residing on human chromosome 5 and not structurally perturbed by the translocation. We validate MRA results for SDH-loss and VHL-loss tumors using gene expression data from a second large cohort of PPGL specimens. These validated lists of subtype-specific MRs may guide the future development of molecularly-targeted therapies for PPGL.

Finally, we generate transcriptomic signatures for SDH loss in cultured mouse embryonic fibroblasts (MEF) via RNA-seq, and perform master regulator analysis on this transcriptomic signature, leveraging a MEF-specific transcriptional network assembled from public data. We then compare lists of SDH-loss MRs inferred in human PPGL tumors and in MEFs to infer MRs conserved between species and cell types. This analysis of conserved SDH-loss MRs in human tumors and MEFs inferred ZNF423/ZFP423, a known modulator of retinoic acid response in neuroblastoma. Functional analysis revealed a blunted cell death response to retinoic acid in SDH-loss MEFs and attenuated neuronal differentiation in SDH-inhibited SH-SY5Y neuroblastoma cells, suggesting a potential developmental etiology for this tumor subtype.

## Materials

### Transcriptomic datasets

RNA-seq gene expression profiles for 179 PPGL specimens (TCGA-PCPG) used to assemble an inferred transcriptional network were obtained via the firebrowse tool available through the Broad Institute. This dataset was described previously in a publication of the TCGA consortium [27].

Microarray data for eight normal adrenal medulla specimens and 44 PPGL tumors used to calculate gene expression signatures for SDH loss and VHL loss were obtained from NCBI GEO accession GSE39716. These data were described previously [41, 42]. Included in this dataset are 13 VHL-null pheochromocytomas, 18 SDHB-null pheochromocytomas and paragangliomas, and 13 SDHD-null pheochromocytomas and paragangliomas, including 8 head and neck tumors. For generation of SDH-loss and VHL-loss gene expression signatures used in the discovery phase of the project, the 18 SDH-null tumors or 13 VHL-null tumors were compared to the 8 normal adrenal medulla specimens.

Microarray gene expression profiles for 188 PPGL specimens comprising the COMETE cohort, used in the MR validation analysis, were obtained from ArrayExpress entry E-MTAB-733. This dataset has been described previously [43, 44]. This dataset includes the following number of tumors with specific known genetic defects: 1 SDHA, 17 SDHB, 2 SDHC, 3 SDHD, 27 VHL, 9 RET, 9 NF1, and 122 tumors of other or unknown genetic cause. For generation of the SDH-loss and VHL-loss gene expression signatures used in the validation phase of this project, the 23 SDH-loss tumors or 27 VHL-loss tumors were compared to the all other PPGL tumors in the cohort.

RNA-seq data used to derive the MEF-specific SDHC-loss transcriptomic signature were generated as described below and deposited in NCBI GEO under accession GSE114244. RNA-seq datasets used to assemble a MEF-specific inferred transcriptional network were obtained from ArrayExpress accessions E-GEOD-72275, E-GEOD-64489, E-GEOD-77351, E-GEOD-79095, E-MTAB-5089, E-GEOD-75631, E-GEOD-68902, E-GEOD-71209, E-MTAB-3875, E-GEOD-70816, E-GEOD-63794, E-GEOD-63756, and from NCBI GEO accession GSE103662.

### Derivation of gene expression signatures

Differential gene expression analysis of COMETE cohort and TCGA-PCPG cohort specimens was conducted using the R package limma, available through Bioconductor. SDH-loss and VHL-loss gene expression signatures were derived by differential gene expression analysis, comparing these specific PPGL tumor subtypes to normal adrenal medulla. Microarray datasets were pre-processed with the application of the robust multi-array average (RMA) algorithm, with downstream differential analysis including linear fitting and analysis of empirical Bayes statistics for differential expression. The *MAML3* translocation gene expression signature used in the discovery phase of the project was derived from TCGA-PCPG data, comparing the 10 identified *MAML3*-mutant tumors to all other tumors (*N* = 169) in the cohort. Similarly, a differential gene expression signature for SDHC-loss in iMEF cells was calculated by performing differential expression analysis on RNA-seq count data, comparing SDHC-null cell lines to hemizygous control lines. The criteria for differential expression signatures in each human PPGL tumor analysis was an absolute log_2_(fold-change) > 1.5 and adjusted *p*-value < 0.05. The criteria for differential expression for SDHC-loss iMEFs was an absolute log_2_(fold-change) > 2.0 and adjusted *p*-value < 0.05.

### Network inference and analysis

Prior to transcriptional network inference (TNI), TCGA RNA-seq data were log_2_-transformed. Transcriptional network inference and master regulator analysis (MRA) were conducted using the R package RTN, as previously described [34], re-implementing other previously-described algorithms [33, 40]. Following transcriptional network inference, data processing inequality filtering (DPI tolerance set to 0 or 0.05) was applied to remove the weakest among inferred interactions prior to master regulator analysis. Each differential expression signature was analyzed separately via MRA to infer putative transcriptional drivers of the observed signatures.

Prior to TNI on MEF RNA-seq data to generate a MEF-specific transcriptional network, raw FASTQ datasets were downloaded from public repositories and aligned to the mm9 genome using the HISAT2 fast read aligner. SAM files yielded from the alignment step were converted to BAM format using Samtools [45], and FPKM gene expression values calculated using R package systemPipeR [46]. Following these steps, FPKM values were log_2_-transformed prior to TNI, as described above for the human PPGL tumor data. Following TNI, MRA was performed using the SDHC-loss MEF gene expression signature to infer transcriptional drivers of the observed signature.

### Network validation

Known motif position-weight matrices for inferred master regulator DNA binding sequences were downloaded from HOMER (http://homer.ucsd.edu/homer/) or JASPAR (http://jaspar.genereg.net/) motif databases. The unfiltered regulon for a subset of human PPGL master regulators was searched for nearest motif match to the transcription start site (TSS) within a 50-kbp window. This search was then repeated 200 times for a scrambled version of the same motif. For each motif search, the fraction of motif matches localizing within 2.5 kbp of the TSS was calculated, and the distribution of scrambled motif fraction compared to the observed fraction for the original motif via calculation of an empiric *p*-value.

### iMEF cell culture and RNA-seq

Cell culture methods and protocol for RNA-seq gene expression analysis for stable SDHC-loss and hemizygous control iMEF cell lines were as described previously [47]. Following high-throughput sequencing, RNA-seq data was deposited in NCBI GEO under accession GSE114244.

### iMEF SOX11 and ZFP423 immunostaining

Stable SDHC-loss and hemizygous control cell lines were plated into 96-well plates at a density of approximately 20,000 cells per well in 100 μL of DMEM medium containing 10% FBS, penicillin (100 units/mL), streptomycin (100 μg/mL), 1 mM pyruvate, 1X MEM NEAA, and 10 mM HEPES buffer (pH 7.2–7.5) and cultured at 21% O_2_ and 5% CO_2_. The next day, medium was aspirated and cells were washed with 100 μL PBS, fixed with 100 μL 3.7% formaldehyde in 1XPBS for 20 min at room temperature, permeabilized with 100 μL 0.1% Triton X-100 in PBS for 15 min at room temperature, and blocked for 15 min at room temperature in PBS containing 10% FBS. Solutions of primary antibody were prepared by dilution of anti-SOX11 (Abcam cat# ab134107) or anti-ZFP423 (Abcam cat# ab94451) primary antibodies 1:100 into PBS containing 10% FBS. Primary antibody solutions were added to plates and incubated for 1 h at room temperature with gentle agitation on an orbital shaker. Wells were then washed 3X with 100 μL PBS, and then incubated for 30 min at room temperature with goat anti-rabbit IgG Alexa Fluor 594 (Molecular Probes cat# R37117) diluted 2 drops per mL into PBS containing 10% FBS. Cells were then washed 3X with 100 μL PBS, and then counterstained with DAPI (5 μg/mL in PBS) for 5 min prior to imaging on a Zeiss LSM 780 confocal microscope using a 10X objective. For each visual field, an autofocus routine was implemented to capture the plane with the highest DAPI staining intensity. Automated image analysis was then employed to quantify compartment-specific immunostaining patterns, as previously described [48].

### Testing effects of retinoic acid in SDHC-loss iMEF lines

Stable SDHC-loss and hemizygous control cell lines were plated into 96-well plates at a density of approximately 10,000 cells per well in 100 μL medium as described above. All-trans retinoic acid (ATRA; Sigma Aldrich cat# R2625) solutions in DMSO were then added to the plated cells to a final concentration of between 78 nM and 5 μM, with final DMSO concentration of 1% of final medium volume. Plates were tapped gently to mix, and then cultured at 21% O_2_ and 5% CO_2_ for 4 d. On the fourth day, the medium and retinoic acid solutions were replaced, at which point phenol red was omitted from the medium, and cells allowed to grow for an additional 2 d under the same conditions. On the 6th day, 10 μL Alamer Blue (Thermo Fisher) cell viability reagent was added to plates and incubated for 6 h prior to taking absorbance measurements at 570 and 600 nm. Cell viability was calculated from Alamar Blue reduction as described previously [47].

### Testing effects of retinoic acid in SH-SY5Y cells

SH-SY5Y neuroblastoma cells were plated into 96-well plates at a density of approximately 15,000 cells per well in 100 μL of DMEM/F12 medium containing 10% FBS, penicillin (100 units/mL), and streptomycin (100 μg/mL) and grown overnight to allow for cell attachment. The next day, diethyl malonate (Sigma Aldrich cat# W237507) stock solutions prepared in ethanol at 500 mM concentration were added to the plate to 5 mM final concentration, and the cells returned to the incubator overnight. Equal volume of ethanol as vehicle control was added to untreated cells. The next day, fresh stock solutions of all-trans retinoic acid (Sigma Aldrich cat# R2625) were prepared in DMSO at 1.2 mM concentration and added to cells to final concentration of 12 μM. Equal volume of DMSO was added to vehicle-treated cells. Cells were returned to the incubator for 2 days to allow for initiation of retinoic acid-induced neuronal differentiation. Cells were then trypsinized (30 μL trypsin), diluted into 500 μL media, and transferred to a poly-D-lysine-coated glass coverslip. Diethyl malonate, retinoic acid, and appropriate vehicle control volumes were then added to previous concentrations and the cells returned to the incubator overnight to allow for cell attachment. The next day, media was aspirated and cells washed with 1 mL PBS, fixed with 3.7% formaldehyde in PBS for 10 min at room temperature, and permeabilized with 0.1% Triton X-100 in PBS for 15 min at room temperature. Cells were the stained with DAPI (Roche cat# 10236276001) and ActinRed 555 ReadyProbes reagent (Invitrogen cat# R37112). Cells were then covered in PBS and imaged by confocal microscopy. Cell morphology of actin staining was then analysed using the MorphologicalSkeleton and MeasureObjectSkeleton subroutines in CellProfiler.

## Results

### Derivation of gene expression signatures

**Fig. 1:**
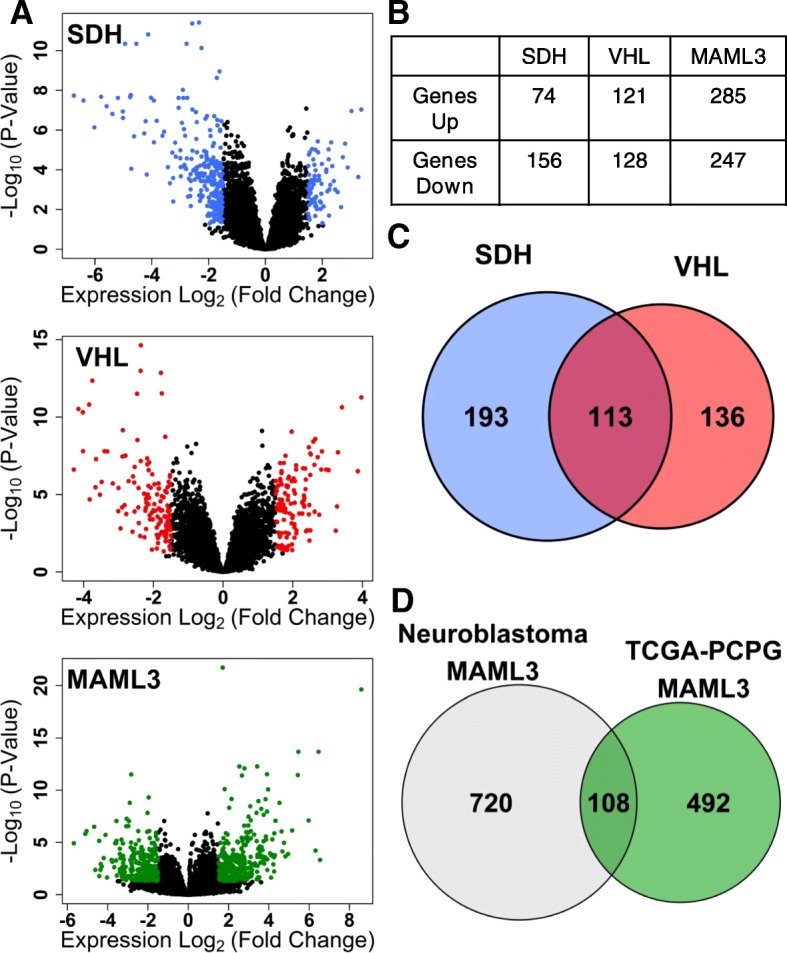
PPGL subtype-specific gene expression signatures. **a** Volcano plots highlighting genes identified in differential expression analysis as having absolute log_2_-fold change > 1.5 and adjusted *p*-value < 0.05. Comparisons for SDH*-*loss and VHL-loss are in reference to normal adrenal medulla. Comparison for *MAML3* translocation is in reference to all other PPGL molecular subtypes. **b** Numbers of differentially-expressed genes detected for each PPGL subtype. **c** Venn diagram showing overlap of differentially-expressed genes detected in SDH-loss, VHL-loss, and *MAML3* transcriptomic signatures. **d** Venn diagram showing overlap of *MAML3* translocation gene expression signatures in PPGL tumors (TCGA-PCPG) and in neuroblastoma tumors

To infer transcription factors responsible for gene dysregulation in PPGL subtypes, signatures of differentially-expressed genes were first derived to seed the MR inference algorithm (MARINa) [40]. We derived these signatures [absolute log_2_(fold-change) > 1.5 and *P*-Value < 0.05] for SDH-loss, VHL-loss, and *MAML3* translocation PPGL subtypes, as described in Methods (Fig. 1a). In total, the identified signatures consist of 230 (SDH-loss), 249 (VHL-loss), and 532 (*MAML3* translocation) differentially-expressed genes (Fig. 1b; Additional file 1: Dataset S1, Additional file 2: Dataset S2, and Additional file 3: Dataset S3). A pattern of general gene expression down-regulation was evident for the SDH-loss signature, with 68% of genes being down-regulated. Approximately equal numbers of genes were up- and down-regulated in each of the VHL-loss and *MAML3* translocation signatures. Interestingly, the gene sets differentially expressed in the SDH-loss and VHL-loss are highly overlapping (empiric *p*-value: 5E-136), compared to the expected overlap for randomly-selected gene sets of this same size (empiric *p*-value 4E-7; Additional file 12: Figure S1). This high degree of overlap between SDH-loss and VHL-loss signatures is consistent with the hypothesis that both tumor molecular subtypes activate pseudohypoxic signalling [49]. We also examined the degree of overlap between the *MAML3* translocation differential gene expression signature for PPGL and that previously observed in *MAML3* translocation-positive neuroblastoma tumors [50] (Fig. 1d), and identify a significant similarity (empiric *p*-value: 3E-35; Additional file 13: Figure S2). This suggests that *MAML3* translocation has similar downstream impacts upon gene expression in these highly similar tumor types.

### Transcriptional network inference and master regulator (MR) analysis

We then assembled a PPGL-specific inferred transcriptional network using 179 RNA-seq experiments generated by the TCGA Research Consortium (http://cancergenome.nih.gov/). This dataset has been described previously [27]. As described in Methods, we used these data to assemble inferred transcriptional networks modeling gene regulation in PPGL tumors, using an implementation of the ARACNE transcriptional network inference (TNI) algorithm available in the R package RTN [34]. We subsequently validated the structure of the inferred transcriptional network by searching the promoter sequences (50 kbp window centered on TSS) of the inferred transcription factor (TF) regulons (i.e. sets of target genes) for known TF DNA-binding domain motifs, extracting the nearest pattern match for each TSS, and comparing to the same searches performed iteratively for scrambled versions of the same motif. We then assessed the fraction of nearest pattern matches for each search localizing to within 2.5 kbp of the TSS. In each of the examined cases, we find that the fraction of motif pattern matches in this window is higher for the original motif than for most scrambled versions (Additional file 14: Figure S3), suggesting that the inferred transcriptional network successfully models real gene-regulatory interactions.

**Fig. 2:**
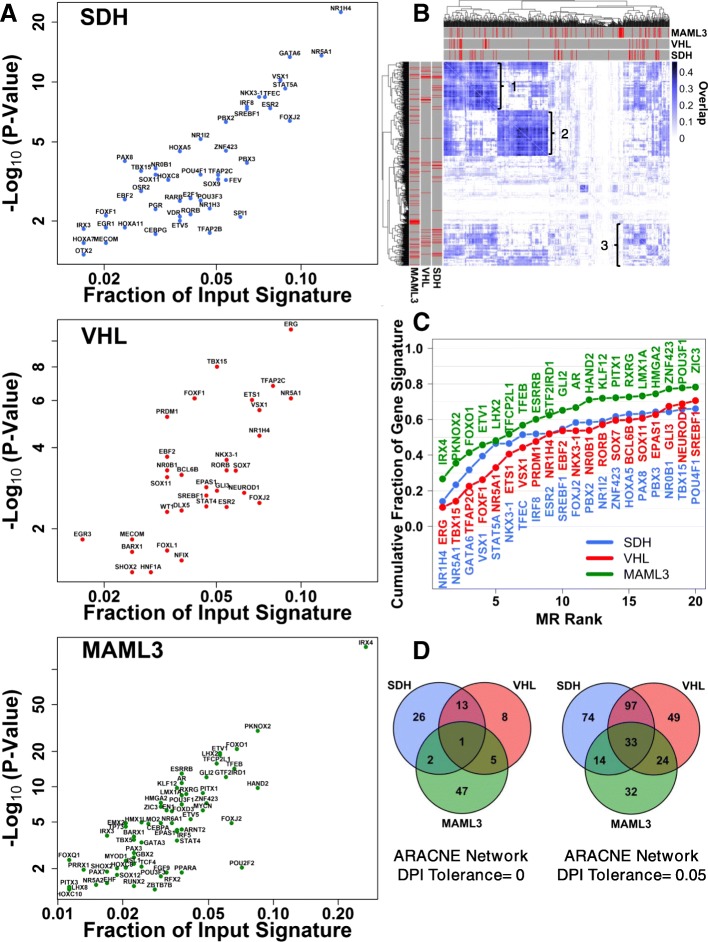
MRs of PPGL transcriptomic signatures. **a** x-y plots showing statistical significance (y-axis) and potential fraction of the input gene expression signature (x-axis) explained by the inferred MRs for each PPGL molecular subtype. **b** Hierarchical clustering analysis of all TFs sorted according to PPGL tumor regulon overlap. Highly co-regulated transcriptional subnetworks are indicated with numeric values. Annotations in red indicate the identities of putative MRs inferred for each PPGL molecular subtype. **c** Running cumulative MR-attributable fraction of SDH-loss, VHL-loss, and *MAML3* translocation gene expression signatures. MRs are ranked in increasing order of MRA *p*-value. **d** Analysis of MR overlap between SDH-loss, VHL-loss, and MAML3 translocation PPGL subtypes for MRA performed on ARACNE inferred transcriptional networks trimmed with DPI tolerance of 0 and 0.05, respectively

We then applied the MR inference algorithm (MARINa; also implemented in R package RTN), using the inferred transcriptional network, to infer TFs whose target genes are significantly enriched for the input SDH-loss, VHL-loss, or *MAML3*-translocation gene expression signatures. First, we performed MRA on ARACNE-inferred transcriptional networks stringently trimmed to remove all but the strongest inferred regulatory connections (DPI tolerance = 0). The results of these analyses, presented in Additional file 4: Dataset S4, Additional file 5: Dataset S5, and Additional file 6: Dataset S6, nominate several dozen high confidence TFs whose target genes are perturbed in these specific PPGL molecular subtypes, with each TF typically controlling between 2 and 10% of differentially-expressed genes. This TF subset nominated by MRA with adjusted *p*-value < 0.05 are shown in Fig. 2a, with the degree of statistical significance from the MRA indicated on the y-axis, and the fraction of the input gene expression signature attributed to each MR indicated on the x-axis.

Surprisingly, hypoxia-inducible factors were not among the nominated high confidence SDH-loss MRs, although HIF2α (encoded by *EPAS1*) was nominated as a MR for VHL-loss tumors, controlling ~ 5% of the observed differentially-expressed genes in that tumor subtype. We therefore repeated the MRA procedure using ARACNE-inferred networks trimmed with slightly less stringent criteria (DPI tolerance = 0.05), so as to enhance the sensitivity of detection of master regulators. These analyses, presented in Additional file 7: Dataset S7, Additional file 8: Dataset S8, and Additional file 9: Dataset S9, reveal evidence of some enhanced *EPAS1*-related transcriptional effects in SDH-loss tumors, although *EPAS1* is by no means among the top MRs inferred to cause observed patterns of tumorigenic transcriptional perturbation. Importantly, this suggests that HIF-related transcriptional dysregulation is detectible, but that it inadequately accounts for the majority of transcriptional perturbations observed in these “pseudohypoxic” tumor subtypes, and that dysregulation of other transcription factors may play a much more important role in driving oncogenic transcriptomic patterns than previously appreciated. Strikingly, for *MAML3* translocation-positive tumors, we find that a single TF, IRX4, is predicted to account for > 20% of the observed transcriptional perturbation. This is particularly intriguing because the reported *MAML3* translocations involve chr18~chr4 fusion (*TCF4~MAML3* gene fusion) or chr17~chr4 fusion (*UBTF-MAML3* gene fusion), with the *IRX4* locus on chromosome 5 being unaffected in either case [27]. It is intriguing and currently unknown how *MAML3* translocation is connected to IRX4-mediated transcriptional dysregulation.

Next, we examined the structure of the inferred PPGL transcriptional network to determine whether the inferred subtype-specific MRs specifically co-regulate common sets of target genes. We therefore calculated in a pairwise fashion the degree to which the regulon (i.e. set of target genes) of a given TF overlaps with the regulon of other known TFs (Additional file 10: Dataset S10). This pairwise matrix of regulon overlap values was then ordered using an unbiased hierarchical clustering algorithm to identify groups of TFs with highly overlapping sets of target genes. This data, presented in Fig. 2b, shows that TFs in PPGL tumors exert their activities on two distinctly different and highly co-regulated subnetworks (labelled #1 and #2, respectively), with an identifiable, but somewhat less co-regulated third subnetwork (labelled #3). Intriguingly, SDH-loss and VHL-loss MRs exert their activities almost exclusively on subnetworks #1 and #3, while sparing subnetwork #2. This asymmetry in the transcriptional subnetworks impacted by PPGL MRs suggested to us the possibility that transcriptional subnetwork #2 is essential for cell survival.

This hypothesis was indeed borne out by more detailed functional analysis of putative transcription-regulatory interactions. We considered the subset of genes inferred to be jointly controlled by at least two transcription factors within a given subnetwork and assessed enrichment for specific gene ontologies. Strikingly, this revealed that each of the three identified subnetworks is highly enriched for specific transcriptional programs (Additional file 14: Figure S3G-I). Subnetwork #1 was found to be highly enriched for genes involved in plasma membrane organization, extracellular matrix (ECM) organization, and regulation of cell migration (Additional file 14: Figure S3G). Subnetwork #3 was found to be highly enriched for genes expressed in the endoplasmic reticulum and various metabolic processes (Additional file 14: Figure S3I). In contrast, subnetwork #2 was found to be highly enriched for nuclear proteins, splicing factors, and proteins involved in ubiquitin-dependent catabolic processes (Additional file 14: Figure S3H). These data support the notion that subnetwork #2 is likely important for cell survival, and additionally, that modulation of subnetwork #1 may be a mechanism by which tumor cells acquire an invasive phenotype. Rigorous testing of these specific hypotheses will be a topic of interest for future follow-up.

We then assessed the degree to which the cumulative effects of the top 20 MRs in each tumor subtype can account for the observed PPGL molecular subtype gene expression signatures. This analysis, presented in Fig. 2c, shows that for each tumor subtype, the top 20 MRs are predicted to account for > 60% of the input tumor subtype-specific gene expression signatures. This suggests that the majority of transcriptomic perturbation for these tumor subtypes may be explicable in terms of the cumulative effects of multiple dysregulated MRs.

Next, we assessed the degree to which the MRs nominated for SDH-loss, VHL-loss and MAML3 translocation-positive PPGL tumors are similar. We therefore assessed the overlap between master regulator lists for these tumor subtypes. This analysis revealed significant overlap between SDH-loss and VHL-loss tumors (Fig. 2d), with a degree of overlap is larger than would be expected by chance considering the size of the specific nominated MR lists (empiric *p*-value: 9E-8; Additional file 15: Figure S4). This suggests an aspect of similarity in transcription factor dysregulation in SDH-loss and VHL-loss PPGL tumors.

### Validation of nominated PPGL subtype-specific MRs

**Fig. 3:**
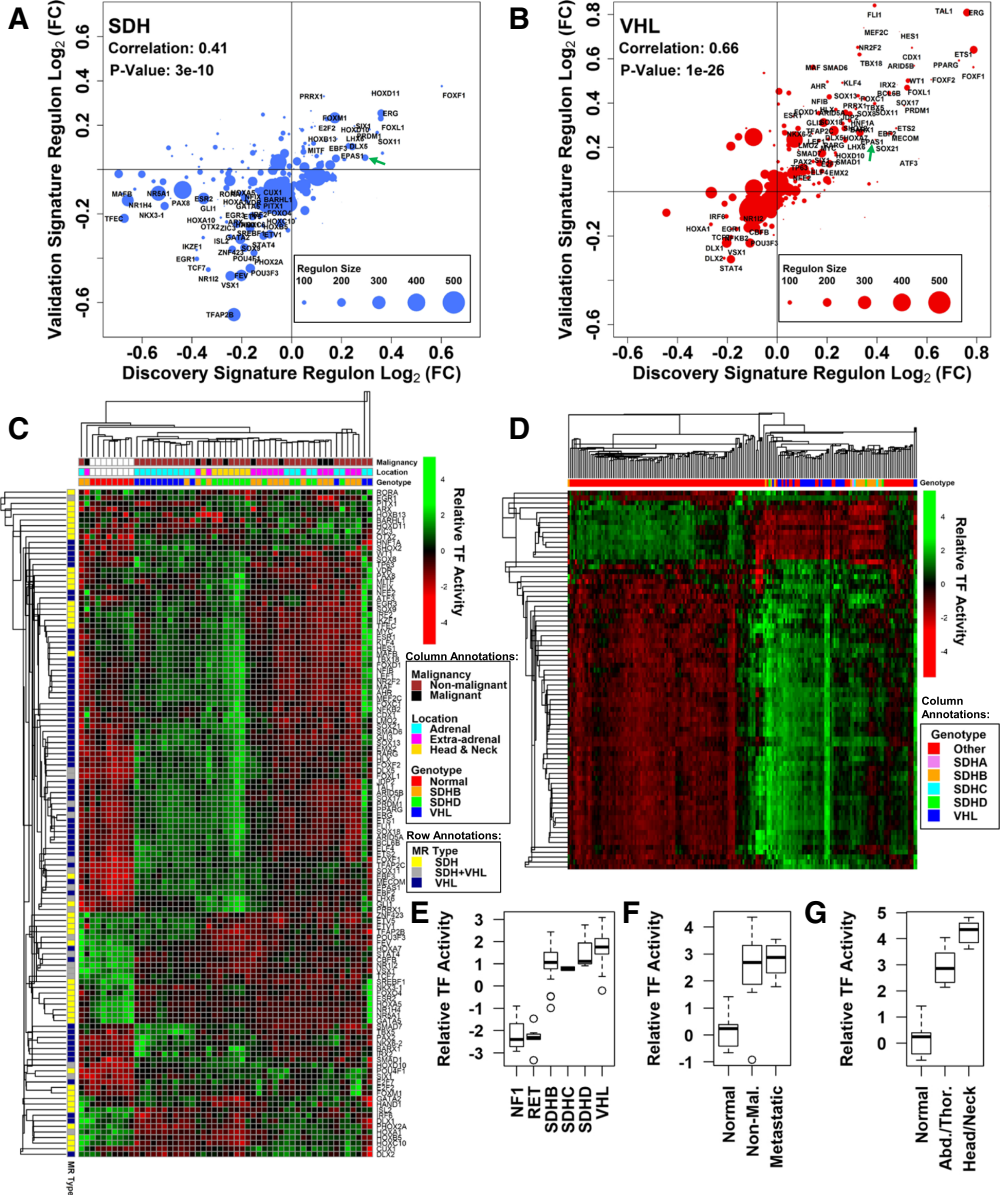
Validation analysis of inferred PPGL subtype-specific MRs. **a** Validation analysis for SDH-loss MRs. Mean inferred regulon log_2_(fold-change) for discovery SDH-loss transcriptomic signature is shown on the x-axis. The same quantification performed for a SDH-loss transcriptomic signature derived from an independent PPGL cohort is shown on the y-axis. Regulon size (number of genes) is indicated by the relative size of the data points. **b** Validation analysis for VHL-loss MRs. Analysis method is the same as in panel (**a**). **c** Hierarchical clustering of discovery cohort PPGL specimen MR transcription factor activity profiles inferred by the VIPER algorithm. Color bars indicate specimen characteristics and MR type, as shown. **d** Hierarchical clustering of validation cohort specimen MR transcription factor activity profiles. Color bars indicate specimen characteristics, as shown. **e** EPAS1 activity for RET, NF1, SDHB, SDHC, SDHD, and VHL specimens in the validation cohort. **f** EPAS1 activity for discovery cohort normal adrenal medulla specimens and SDHB-null PPGL tumors annotated as “metastatic” or “non-malignant”. **g** EPAS1 activity of discovery cohort normal adrenal medulla specimens and SDHD-null PPGL tumors of the head and neck and those arising from abdomen and thorax

Beyond nomination of MRs that collectively explain patterns of dysregulated gene expression in specific PPGL molecular subtypes, we sought to validate the nominated MRs using data from an independent large cohort of PPGL specimens (*N* = 188). This dataset has been described previously [43, 44]. Using known information regarding *SDHx* and *VHL* mutation status of these tumor specimens, we calculated the average log_2_(fold-change) in gene expression for tumors of each of these molecular subtypes relative to other PPGL tumors. Then, using known regulon structures of the PPGL inferred transcriptional network, we calculated the average log_2_(fold-change) for all genes under regulatory control of each of the SDH-loss and VHL-loss MRs nominated from MRA performed on the ARACNE network trimmed with DPI tolerance of 0.05. We performed this same calculation using the input gene expression signature used for MR discovery, and then examined whether there was correlation between the regulon log_2_(fold-change) values for discovery and validation data sets. Strikingly, we observe strong agreement between the two analyses (Fig. 3a, b). The strength of statistical correlation is especially remarkable considering the fact that the discovery signature involves a tumor-normal comparison while the discovery signature involves a tumor-tumor comparison. The validation analysis therefore supports the claim that the inferred dysregulated MR activities are biologically-reproducible phenomena that are consistent across independent patient cohorts.

We pursued further analysis of the discovery and validation cohort data, asking whether these altered TF activities are sufficient to drive hierarchical clustering of PPGL specimens that accurately separates specimens according to molecular subtype. For this analysis, we leveraged the VIPER algorithm, which is capable of robustly converting individual transcriptomic datasets into TF activity profiles [51]. When this algorithm is applied to discovery cohort specimens including SDHB-null, SDHD-null, and VHL-null PPGL tumors from various anatomical locations, as well as normal adrenal medulla specimens, the hierarchical clustering algorithm generally separates normal adrenal samples, VHL-null pheochromocytomas, and SDHD-null head and neck paragangliomas away from the remaining SDHB-null and SDHD-null PPGL tumors (Fig. 3c). This suggests that significant differences in transcription factor activity profiles exist between SDHD-null head and neck PPGL tumors and SDH-null tumors arising from other anatomical locations, although SDH-loss tumors are generally more self-similar than PPGL tumors arising from other genetic defects (Fig. 3d). These patterns were further reproduced by t-SNE clustering (Additional file 16: Figure S5), similarly underscoring the unique TF activity profile of head and neck PPGLs compared to tumors arising from SDH subunit defects in abdomen or thorax, but with little other difference that can be attributed to specific SDH subunit defects.

We therefore pursued a more detailed analysis of differences in TF activity profiles that exist between SDHD-null head and neck PPGL tumors and SDHD-null tumors from abdomen and thorax. This analysis, presented in Additional file 17: Figure S6, reveals several specific differences in transcription factor activity that correlate with anatomical location. Among the most impressive differences in TF activity, head and neck tumors display lower activities of FEV, TFAP2B and GATA2 and higher activities of TFEC, LEF1, and MAFB compared to SDHD-null tumors of other anatomical locations. The basis for this is unclear, but this finding clearly suggests that tumor anatomic location and underlying genotype synergize to drive observed molecular signatures at the transcriptional level. Whether any of these differences is responsible for the generally more benign character of head and neck neoplasms remains to be seen.

We then specifically considered EPAS1, a transcription factor of much interest to the field, testing the hypothesis that tumors originating from different SDH subunit mutations have variable EPAS1 activity. This analysis, presented in Fig. 3e, revealed similarly elevated levels of EPAS1 activity in SDHB-null, SDHC-null, and SDHD-null PPGL tumors compared to those attributable to RET of NF1 gain-of-function mutations. Additionally, EPAS1 activities for all SDHx-loss tumors were roughly similar to those observed in VHL-loss tumors. These data suggest that EPAS1 activity is similarly elevated for all PPGL tumors exhibiting VHL loss or SDH complex bi-allelic loss of function, regardless of the particular SDH subunit involved.

We next tested the hypothesis that EPAS1 activity is significantly different in SDHB-null metastatic tumors vs. those annotated as “non-malignant”. This analysis, presented in Fig. 3f, did not detect any significant differences in EPAS1 activity between metastatic and “non-malignant” tumors. Indeed, an expanded analysis considering the activities of all transcription factors inferred via the VIPER algorithm did not identify a single TF that is differentially active between metastatic SDHB-null tumors and those annotated as “non-malignant”. This suggests either a lack of statistical power in the analysis, or else the lack of a true biological distinction between the involved samples. Also, as a subset of the specific analysis of SDH MR activities in SDHD-null tumors of the head and neck vs. those arising from other anatomical locations, we did not note any statistical difference in EPAS1 activity between tumors arising from these locations (Fig. 3g). Collectively, these data suggest that differences in EPAS1 activity are not appreciably different between SDH-null tumors of variable malignant character or those arising from disparate anatomic locations.

### Analysis of conserved SDH-loss MRs

**Fig. 4:**
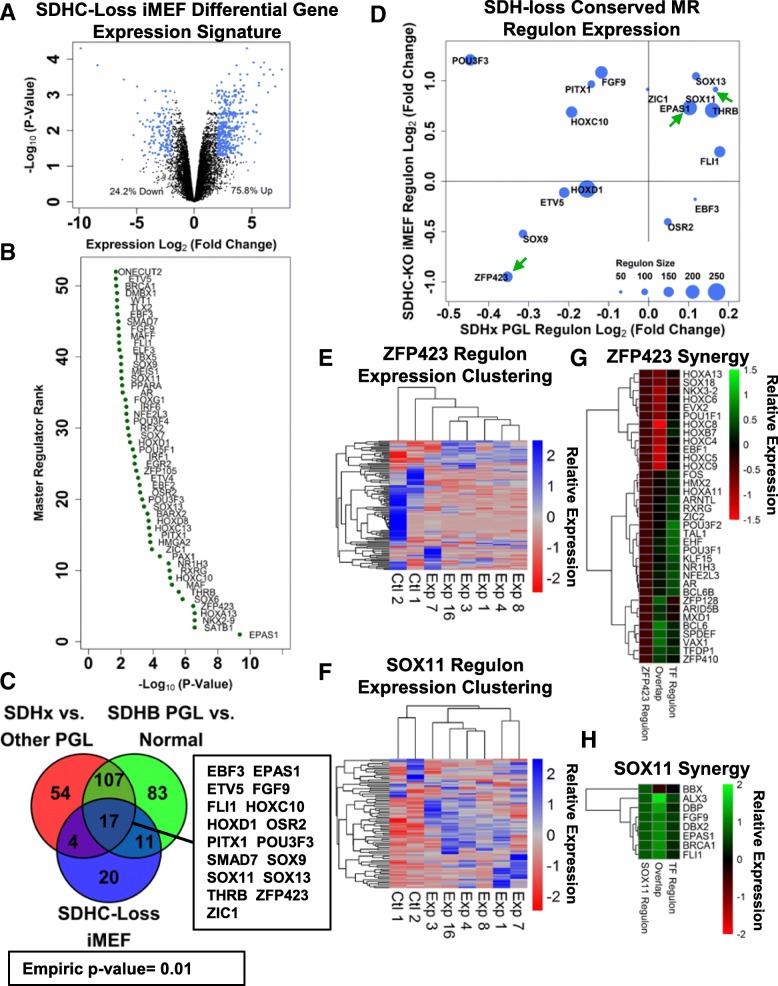
Analysis of conserved SDH-loss MRs. **a** Volcano plot showing SDHC-loss MEF transcriptomic signature. **b** SDHC-loss MEF MRs organized according to MR rank. **c** Venn diagram showing overlap of SDHC-loss MEF MRs and SDH-loss MRs inferred in PPGL human tumors. Green circle shows SDH-loss MRs inferred in the discovery cohort analysis. Red circle shows MRs inferred leveraging the validation cohort SDH-loss gene expression signature. Blue circle shows SDHC-loss iMEF MRs. Empiric *p*-value estimated by analysis of degree of overlap for iteratively-generated randomly-selected sets of TFs is shown. Putative conserved SDH-loss MRs are indicated. **d** Analysis of regulon log_2_(fold-change) for putative conserved SDH-loss MRs. Regulon expression change for human SDH-loss PPGL tumors is shown on the x-axis. Regulon expression change for SDHC-loss MEFs is shown on the y-axis. Average size of mouse and human regulons are indicated by the size of data points. **e** Hierarchical clustering of MEF samples based upon ZFP423 regulon gene expression patterns (Exp: SDHC knockout iMEF line; Ctl: hemizygous control iMEF line). **f** Hierarchical clustering of MEF samples based upon SOX11 regulon gene expression patterns (Exp: SDHC knockout iMEF line; Ctl: hemizygous control iMEF line). **g** Analysis of synergy between ZFP423 and other mouse TFs. Regulon expression for ZFP423 is indicated as the leftmost column. Regulon expression for the various other TFs are indicated as the rightmost column. Regulon expression for the subset of co-regulated genes are shown in the middle column. **h** Analysis of synergy between SOX11 and other mouse TFs. Method of representation is the same as in panel (**g**)

Beyond analysis of SDH-loss MRs in human PPGL tumors, we next asked whether there is any evidence for conservation of SDH-loss MR responses across species and cell types. To answer this question, we generated a transcriptomic signature of SDHC-loss immortalized mouse embryonic fibroblasts (iMEFs). This model of SDHC-loss was used rather than the more common SDHB and SDHD subunit defects seen in human PPGL tumors specifically because the SDHC gene trapped allele used to derive the line was readily available from the Wellcome Trust Sanger Institute Gene Trap resource. Examination of transcriptional patterns in this model revealed general transcriptional up-regulation (Fig. 4a) that is different from that observed in human tumors. The reason for this is unclear. We next performed MR analysis on a MEF-specific inferred transcriptional network, as described in Methods. In total, this approach nominated 73 MR TFs whose target genes are perturbed upon SDHC-loss (Additional file 11: Dataset S11). Notably, *EPAS1* was nominated as the top-ranking SDHC-loss MR gene, in contrast to the SDH-loss human PPGL tumor analysis (Fig. 4b). The reason for this difference is unclear, but it seems likely that cultured cell lines provide homogeneous populations, whereas tumors have significant cellular heterogeneity. In any case, the observation that the top SDHC-loss MEF MR is EPAS1 (HIF2α) lends considerable support to the pseudohypoxia hypothesis of succinate-mediated poisoning of prolyl hydroxylases catalysing HIF factor hydroxylation. This being the case, we emphasize that EPAS1 dysregulation is still inferred to account for only ~ 5% of dysregulated genes in SDHC-loss MEFs, indicating that alternative explanations must be sought to account for the majority of the observed dysregulated gene expression signature. These explanations likely include dysregulation of other TFs, as observed in our analysis.

We hypothesized that although human PPGL tumors and mouse SDH-loss iMEFs are obviously very different, fundamental similarities might point to drivers of PPGL tumorigenesis. We therefore examined the overlap between the inferred SDHC-loss iMEF MRs and MRs inferred via unbiased MR analyses in human SDH-loss PPGL tumors. This analysis revealed five potentially conserved MRs, a number that is higher than would be predicted by random chance, suggesting that perturbation of these MRs represents a conserved biological response to SDH loss (Fig. 4c). We then assessed whether the expression changes for the inferred regulons of each of these MRs in SDH-loss human PPGL tumors and in SDHC-loss MEFs display similar patterns of regulatory perturbation. This analysis, presented in Fig. 4d, reveals that 4 of the 5 conserved SDH-loss MRs also show conserved patterns of regulon gene expression perturbation. These 4 MRs (ZNF423/ZFP423, SOX9, ETV5, and SOX11) thus represent high-confidence conserved SDH-loss MRs. Since SOX11 and ZFP423 exhibit the most dramatic patterns of altered regulation, these were chosen for follow-up analysis.

We examined the patterns of differential gene regulation for the inferred regulons of ZFP423 and SOX11, assessing the degree to which expression of these gene subsets is sufficient to drive unbiased hierarchical clustering of iMEF experimental (SDHC-loss) and hemizygous control specimens (Fig. 4e, f). The results of these analyses show that the regulon for each of these MRs is indeed sufficient to segregate experimental and control cell lines, suggesting that the regulatory activities of these factors are indeed characteristically perturbed in SDHC-loss cell lines. Interestingly, despite down-regulation being the predominant pattern of gene dysregulation for the ZFP423 regulon in SDHC-loss cells, a subset of genes displays consistent up-regulation relative to the control cell lines. This suggested to us the possibility that ZFP423 may not be acting simply as a transcriptional co-activator.

We therefore assessed patterns of gene expression regulatory synergy between ZFP423 and other known TFs to determine whether co-regulation may modulate the transcriptional effects of ZFP423. This analysis involved assessing in a pairwise fashion whether the portion of the ZFP423 regulon overlapping with another TF is differentially-expressed relative to the full ZFP423 regulon, and to the full regulon of the other TF. This analysis, presented in Fig. 4g, revealed three distinct modes of transcriptional modulation by ZFP423, suggesting complicated TF function. For a subset of TFs (HOXA13, SOX18, NKX3–2, HOXC6, EVX2, POU1F1, HOXC8, HOXB7, HOXC4, EBF1, HOXC5, and HOXC9), ZFP423 displays synergistic transcriptional repression. Encouragingly, EBF1 has previously been described as a ZNF423 interaction partner, with synergistic repressive effect, suggesting that the other synergistic repressive partners inferred by this method may also be genuine [52]. A second subset of synergistic partners (FOS, HMX2, HOXA11, ARNTL, RXRG, ZIC2, POU3F2, TAL1, EHF, POU3F1, KLF15, NR1H3, NFE2L3, AR, and BCL6B) was inferred for which ZFP423 binding attenuates the general transcription-activating activity of these TFs. A third subset of synergistic partners (ZFP12B, ARID5B, MXD1, BCL6, SPDEF, VAX1, TFDP1, and ZFP410) was inferred with synergistic activation of gene expression in the co-regulated gene subset. These synergistic activating interactions likely account for the small subset of genes in the ZFP423 regulon that display up-regulation upon SDHC-loss. The complex pattern of gene up- and down-regulation upon SDHC-loss reflects the known complex role of ZFP423 in gene regulation.

**Fig. 5:**
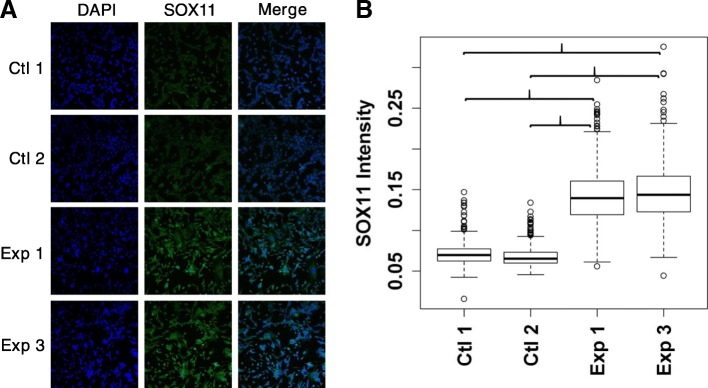
SOX11 immunostaining in SDHC-loss and control MEFs. **a** Representative immunostain images of stable SDHC-loss (Exp) and hemizygous control (Ctl) MEF lines. **b** Quantification of mean cellular SOX11 immunostain intensity using CellProfiler automated image analysis. Comparisons indicated by asterisks are statistically significant by a two-sided heteroscedastic t-test (*p* < 0.05)

Similar analysis of synergistic interactions for SOX11 was simpler, with generally only synergistic transcription-activating interactions detected. Putative synergistic activating partners include ALX3, DBP, FGF9, DBX2, EPAS1, BRCA1, and FLI1 (Fig. 4h). Immunostaining for SOX11 levels in SDHC-loss and control MEFs revealed an increased SOX11 signal in the SDHC-loss context (Fig. 5a, b). Taken together with the general pattern of regulon up-regulation upon SDHC-loss and the sparse synergistic up-regulatory interactions with other factors, this is consistent with SOX11 having a role as a simple transcriptional activator. The reasons why the cellular concentration of SOX11 is increased in SDHC-loss MEFs remain unclear, however.

**Fig. 6:**
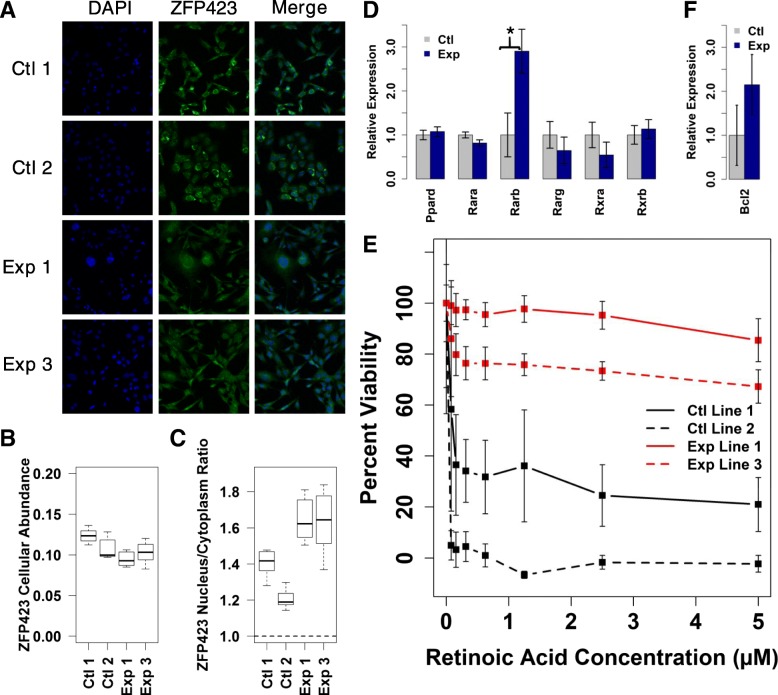
Analysis of ZFP423 and retinoic acid effects in SDHC-loss MEFs. **a** Representative ZFP423 immunostain images of stable SDHC-loss (Exp) and hemizygous control (Ctl) MEF lines. **b** Analysis of ZFP423 mean cellular immunostain intensity using CellProfiler automated image analysis approach. **c** Analysis of ZFP423 subcellular localization using CellProfiler automated image analysis. **d** Relative RNA-seq gene expression quantification for known retinoic acid receptors and transcriptional co-activators. Comparisons indicated by asterisks are statistically significant by a two-sided heteroscedastic t-test (*p* < 0.05). **e** Alamar blue cell viability analysis following 6-d exposure of MEF cells to retinoic acid. **f** Relative RNA-seq gene expression quantification for Bcl2

Interestingly, immunostaining for ZFP423 in SDHC-loss and control MEFs did not reveal dramatic differences in cellular abundance of this MR, suggesting that bulk increase or decrease in cellular ZFP423 protein is not a feature of SDHC-loss (Fig. 6a, b). We did, however, detect generally increased nuclear localization of ZFP423 in SDHC-loss context (Fig. 6c). Understanding the basis for this altered nuclear localization of ZFP423 upon SDHC-loss will require future study.

ZNF423 has previously been described as a transcriptional cofactor of RARα/RXRα that is critically required for retinoic acid-induced differentiation of neuroblastoma [53]. An aspect of this prior work was the demonstration that ZNF423 physically associates with RARα/RXRα in the region of the RARβ promoter, potentially driving transcriptional activation through the RARα/RXRα complex. We therefore examined our RNA-seq gene expression data from SDHC-loss and control MEF lines to determine whether there is any evidence for differential expression among any of the known retinoic acid receptors (RAR) or retinoid X receptors (RXR). Intriguingly, we find that *Rarβ* gene expression is significantly up-regulated in SDHC-loss cells (Fig. 6d). If ZFP423 is a transcriptional cofactor required for activation of *Rarβ* gene expression, increased nuclear localization of ZFP423 upon SDHC-loss and subsequent increased occupancy of the *Rarβ* gene locus would potentially account for the observed transcriptional activation of *Rarβ* expression.

### Retinoic acid resistance of SDH-loss MEFs

The observation that SDHC-loss cells display increased nuclear localization of ZFP423 and concomitant up-regulation of *Rarβ* gene expression suggested to us the possibility of differential cellular response to retinoic acid. We tested this as described in Methods. After 6 d of exposure to retinoic acid over a range of concentrations, we assayed cell viability as a function of genotype. Strikingly, we observe robust induction of cell death in both control cell lines at relatively low concentrations of retinoic acid, but little effect on viability for SDHC-loss lines (Fig. 6e). This suggests that SDHC-loss status fundamentally modulates the cellular response to retinoic acid, one of the most powerful known endogenous morphogens. How perturbation of ZFP423 and *RARβ* results in decreased retinoic acid-induced cell death is unclear. Previous work has suggested that *BCL2* overexpression is predictive of a differentiation response rather over induction of apoptosis in cultured tumor cell lines [54]. Intriguingly, we see that *BCL2* expression is generally up-regulated in SDHC-loss MEF lines relative to controls, although the comparison is not statistically significant (Fig. 6f). Our results suggest that SDHC loss may fundamentally modulate cells away from a death response to retinoic acid.

### Retinoic acid resistance of SH-SY5Y neuroblastoma cells

**Fig. 7:**
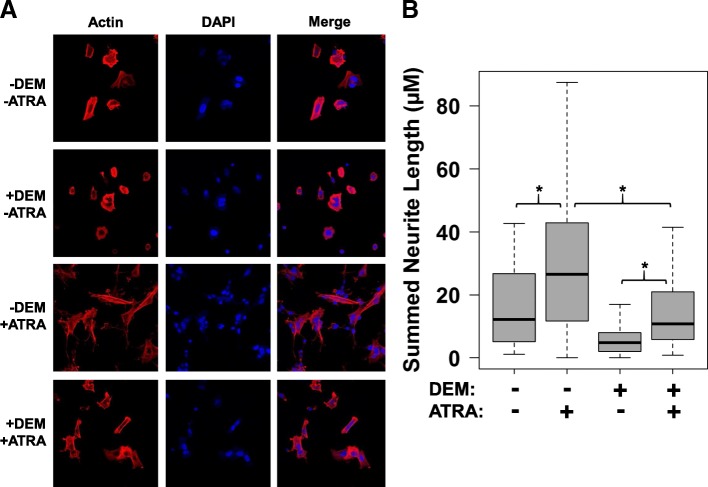
Analysis of SDH inhibition effects upon retinoic acid-induced differentiation of SH-SY5Y neuroblastoma cells. **a** Representative confocal microscopy images of SH-SY5Y cells treated with SDH complex inhibitor diethyl malonate (DEM, 5 mM) and/or all-trans retinoic acid (ATRA, 12 μM), visualized with actin and DAPI staining. **b** Quantification of neurite lengths for individual cells obtained via automated image analysis in CellProfiler (*N* > 40 cells per condition,* Wilcox rank sum *p*-value <1E-4)

Since retinoic acid responses differ between cell types, with the apoptotic responses on MEFs not necessarily predicting the retinoic acid responses of neuronal cells, we therefore decided to test the effects of SDH complex inhibition on the retinoic acid-induced differentiation of SH-SY5Y neuroblastoma cells. The differentiation responses of SH-SY5Y to retinoic acid have been previously well-characterized, with resultant phenotypic changes in neurite outgrowth that are readily apparent through morphological image analysis that has been broadly used to probe the pathways involved in neuronal differentiation [55–58]. We therefore studied the effects of SDH complex inhibitor malonate (formulated as cell-permeable diethyl ester) upon retinoic acid-induced differentiation in the SH-SY5Y neuroblastoma cell line, assessing for changes in neurite outgrowth as a function of exposure to diethyl malonate (DEM) and/or retinoic acid. Intriguingly, we observe that the cellular differentiation response to 12 μM all-trans retinoic acid for 72 h is blunted when cells are pre- and co-treated with 5 mM diethyl malonate (DEM), evident through relatively attenuated neurite outgrowth (Fig. 7). This result aligns with the date from the MEF studies demonstrating that SDH loss attenuates the normal cellular response to retinoic acid, suggesting that attenuated retinoic acid response may be a generalized property of cells lacking SDH complex activity. How these phenomena relate to human PPGL tumors remains to be seen.

## Discussion

Here we apply well-validated TNI and MRA methods to infer the main transcriptional drivers responsible for the observed patterns of differential gene expression in SDH-loss, VHL-loss, and *MAML3*-translocation PPGL subtypes. Using calculated gene expression signatures, we show that application of TNI/MRA nominates sets of MRs that collectively explain the majority of the gene expression perturbation observed in these PPGL tumor subtypes. This, in turn, suggests that perturbation of TF activities plays a larger role in modulating oncogenic gene expression in these tumors than previously appreciated.

Considering the sets of nominated MRs, we find a high degree of overlap between SDH-loss and VHL-loss PPGL subtypes. This suggests a common mechanism of TF perturbation that is similar in both molecular PPGL subtypes. Since the molecular defect in VHL-loss tumors is mutation of the VHL E3 ubiquitin ligase downstream of the prolylhydroxylases believed to be poisoned by succinate accumulation, one potential hypothesis is that these conserved MRs are previously unappreciated direct or indirect clients of both prolylhydroxylases and VHL E3 ubiquitin ligase. In principle, this hypothesis could be tested. Other potential explanations for this overlap in MRs may also exist.

A virtue of the analysis reported here is that it has high clinical potential because it is derived from human PPGL gene expression profiles. For example, the finding that the *IRX4* gene has inferred regulatory control of over 20% of perturbed genes inferred in the *MAML3* translocation PPGL subtype makes follow-up of this observation an important priority. Indeed, for all inferred MRs for each of the three PPGL signatures, eventual experimental pursuit of these relationships will be needed to judge the potential clinical utility of these findings. However, significant challenges currently exist for experimental validation of the observations reported here. No relevant PPGL mouse models or PPGL tumor cell lines are available, making confirmatory live cell experiments impossible. When such tools become available, perturbation experiments will be required to ask whether the inferred MRs are targets for potential therapeutic intervention.

Validation of putative SDH-loss and VHL-loss MRs was possible due to the availability of public transcriptomic datasets that could be leveraged to calculate independent gene expression signatures for these molecular subtypes. This validation set was then leveraged to evaluate the consistency of putative MR effects. Unfortunately, no such validation dataset is currently available for *MAML3* translocation-positive PPGL tumors. The MRs inferred in that portion of the analysis therefore remain putative. For SDH-loss and VHL-loss analyses, remarkable consistency was observed in regulon perturbation between discovery and validation datasets. This indicates that the altered MR activities detected in the discovery analysis are biologically reproducible phenomena.

Classical role of pseudohypoxic activation of gene expression via HIF factors in SDH-loss and VHL-loss PPGL tumors has not previously been studied via unbiased methods such as TNI/MRA. Our MR discovery analysis of human PPGL tumors inferred *EPAS1* perturbation in both SDH-loss and VHL-loss tumors, but the overall contribution to tumorigenic transcriptional patterns in both tumor types is underwhelming. The reason for this is unclear. Analysis of SDHC-loss MEF transcriptomic signature via TNI/MRA did, however, infer EPAS1 (HIF2α) as a MR, lending strong support to the pseudohypoxia hypothesis of prolyl hydroxylase dioxygenase poisoning by accumulated succinate. Nonetheless, a key conclusion from our analysis is that perhaps only 5% of differentially-expressed genes are controlled by HIF2α in these pseudohypoxic tumors, suggesting that other mechanisms must be invoked to explain the remaining 95% of observed transcriptional dysregulation. Categories of other potential mechanisms include dysregulation of other TFs, classical epigenomic derangement via dioxygenase poisoning of TET DNA demethylases and Jumonji domain-containing histone demethylases, as well as dysregulation of cellular acylation patterns, as recently described [59].

Our analysis of MRs consistently perturbed in both human SDH-loss PPGL tumors and in SDHC-loss MEFs suggests that at least a portion of the MR response to SDH loss is conserved among vertebrates. Functional analysis of ZFP423, the mouse ortholog of human ZNF423, suggested that SDHC-loss MEFs might have a differential cellular response to retinoic acid. Our experiments show that this is indeed the case. We observe that control immortalized MEFs respond to retinoic acid by cell death, most likely through induction of apoptosis. Remarkably, this induced cell death upon retinoic acid exposure is not observed in SDHC-loss cells. We additionally show that malonate-mediated inhibition of SDH activity in the SH-SY5Y neuroblastoma cell line inhibits the normal process of retinoic acid-induced neuronal differentiation. These results suggest that SDH loss or inhibition may fundamentally modulate the cellular response to retinoic acid, one of the most potent known endogenous morphogens. The provocative extension of this observation is speculation that SDH-loss PPGL tumors may originate in development through a failed apoptotic response to retinoic acid concentration gradients. This concept of a putative developmental origin for SDH-loss PPGL through failure to properly interpret apoptotic cues has been previously raised in the context of neuronal growth factor signalling [60]. Intriguingly, this is not the first indication that retinoic acid responses may be altered in SDH-loss paragangliomas. Previous reports have suggested that expression of retinol binding protein 1 (RBP1) is attenuated in SDH-null paragangliomas relative to PPGL tumors of other genotypes [61]. We also observe that this is the case (Additional file 18: Figure S7). Whether attenuated apoptotic response to retinoic acid is a feature of SDH-loss PPGL tumors remains to be confirmed.

## Conclusions

We present here unbiased analyses nominating specific MR TFs that collectively explain the observed patterns of transcriptomic perturbation in SDH-loss, VHL-loss, and *MAML3* translocation-positive PPGL tumors. Our analyses generally confirm the accepted mechanism of pseudohypoxic activation of EPAS1/HIF2α in SDH-loss and VHL-loss contexts, but suggest that this effect accounts for only ~ 5% of differential gene expression, leaving the vast majority of tumorigenic transcription aberrations to be explained. Many of the other nominated MRs currently lack clear mechanistic connections to their primary gene defects, but show characteristic and consistent patterns of perturbation across tumor specimens. Future investigation may help to elucidate the relevant mechanistic details.

We also present analysis of MRs inferred in an SDHC-loss MEF tissue culture model that suggests that a subset of the SDH-loss MR response is conserved between vertebrate species and across cell types. Subsequent analysis of one of the conserved MRs, ZFP423, suggests that altered response to retinoic acid may be a distinguishing feature of SDHC-loss MEFs, a hypothesis that we tested and validated experimentally. We report that SDHC-loss MEFs display an attenuated cell death response to retinoic acid and that SDH-inhibited SH-SY5Y neuroblastoma cells display attenuated retinoic acid-induced neuronal differentiation. This retinoic acid resistance suggests a possible developmental path to SDH-loss PPGL tumorigenesis.

## Additional files

Additional file 1: **Dataset S1.** SDHB-loss PPGL differential gene expression signature. (CSV 14 kb)

Additional file 2: **Dataset S2.** VHL-loss PPGL differential gene expression signature. (CSV 11 kb)

Additional file 3: **Dataset S3.** MAML3 translocation-positive PPGL differential gene expression signature. (CSV 19 kb)

Additional file 4: **Dataset S4.** SDHB-loss PPGL master regulators (dpi = 0.00). (CSV 18 kb)

Additional file 5: **Dataset S5.** VHL-loss PPGL master regulators (dpi = 0.00). (CSV 18 kb)

Additional file 6: **Dataset S6.** *MAML3* translocation-positive PPGL master regulators (dpi = 0.00). (CSV 19 kb)

Additional file 7: **Dataset S7.** SDHB-loss PPGL master regulators (dpi = 0.05). (CSV 22 kb)

Additional file 8: **Dataset S8.** VHL-loss PPGL master regulators (dpi = 0.05). (CSV 22 kb)

Additional file 9: **Dataset S9.** *MAML3* translocation-positive PPGL master regulators (dpi = 0.05). (CSV 21 kb)

Additional file 10: **Dataset S10.** PPGL regulon overlaps. (CSV 3893 kb)

Additional file 11: **Dataset S11.** SDHC-loss iMEF master regulators. (CSV 19 kb)

Additional file 12: **Figure S1.** Statistical analysis of differential expression gene set overlaps for SDH-loss and VHL-loss PPGL tumor molecular subtypes. Statistical simulations assessing the probability of the observed differential expression gene set overlaps. Gray histogram bars show the distribution of overlaps for randomly-selected gene sets of the same size as those analyzed. Green dots and lines show Poisson fit to the simulated data and estimated *p*-value for the observed overlap relative to the simulated overlap distribution. (PDF 156 kb)

Additional file 13: **Figure S2.** Statistical analysis of differential expression gene set overlaps for *MAML3* translocation-positive PPGL tumors and *MAML3* translocation-positive neuroblastoma tumors. Statistical simulations assessing the probability of the observed differential expression gene set overlaps. Gray histogram bars show the distribution of overlaps for randomly-selected gene sets of the same size as those analyzed. Green dots and lines show Poisson fit to the simulated data and estimated *p*-value for the observed overlap relative to the simulated overlap distribution. (PDF 73 kb)

Additional file 14: **Figure S3.** PPGL transcriptional network validation by analysis of known TF-binding DNA motifs in inferred MR regulons and analysis of transcriptional subnetworks. A,C,E) Red traces show distribution of nearest pattern match for known TF-binding DNA motifs in inferred TF regulon. Gray traces show average nearest pattern match for scrambled version of the same motif. B,D,F) Statistical analysis of regulon motif pattern searching. Red dot indicates the fraction of nearest pattern matches for the original motif localizing to within 2.5 kbp of the TSS. Gray bars show the distribution of values yielded from the same quantification performed on scrambled versions of the original motif. Empiric *p*-values were estimated from the data in the random distribution and expected likelihood of the observed motif fraction with 2.5 kbp of the TSS. G-I) Analysis of transcriptional subnetwork-specific functional term enrichment among inferred target genes. Shown are the top 10 gene ontologies and/or KEGG pathways unique to each subnetwork. Subnetworks refer to those specified in Fig. 2b. (PDF 470 kb)

Additional file 15: **Figure S4.** Statistical analysis of master regulators inferred in SDH-loss and VHL-loss PPGL tumors. Statistical simulations assessing the probability of the observed differential expression gene set overlaps. Gray histogram bars show the distribution of overlaps for randomly-selected gene sets of the same size as those analyzed. Green dots and lines show Poisson fit to the simulated data and estimated *p*-value for the observed overlap relative to the simulated overlap distribution. (PDF 85 kb)

Additional file 16: **Figure S5.** t-SNE clustering of PPGL tumors by inferred transcription factor activity profile. A) t-SNE clustering of discovery cohort PPGL tumors by transcription factor activity profile. Colors of the data points correspond to annotations for tumor genotype, malignancy, and location, as indicated. B) t-SNE clustering of COMETE validation cohort PPGL tumors by transcription factor activity profile. Colors of the data points correspond to annotations for tumor genotype, as indicated. C) Assessment of EPAS1, ZNF423, and SOX11 activities in validation cohort specimens. Clustering pattern corresponds to genotype annotations given in panel B. (PDF 150 kb)

Additional file 17: **Figure S6.** Analysis of transcription factor activity profiles of SDHD-null head and neck tumors. A) Analysis of differential SDH-loss PPGL master regulator activity in SDHD-null tumors of the abdomen and thorax vs. head and neck tumors. X-axis indicates log_2_(fold change) in inferred transcription factor activity between tumors of the and thorax relative to head and neck tumors. Y-axis indicates degree of statistical significance for the comparison. The subset of data with adjusted *p*-value < 0.05 are plotted in green and include a text label. B-E) Boxplots showing distribution of activity profiles for selected differentially active SDH-loss MRs. (PDF 174 kb)

Additional file 18: **Figure S7.** Analysis of RBP1 expression in validation cohort PPGL specimens. A-B) t-SNE clustering of COMETE validation cohort PPGL tumors by transcriptional profile. Colors in panel A indicate relative degree of RBP1 expression (red = low, blue = high). Colors in panel B correspond to annotations for tumor genotype, as indicated. (PDF 197 kb)

## Data Availability

The RNA-seq datasets generated in the current study are available in the NCBI GEO repository under accession GSE114244. All other datasets analyzed in this work are public domain, as described in Methods.

## Abbreviations

MAML3: Mastermind like transcriptional coactivator 3
MR: Master regulator
MRA: Master regulator analysis
PPGL: Paraganglioma and pheochromocytoma
SDH: Succinate dehydrogenase
SDHB: Succinate dehydrogenase subunit B
SDHC: Succinate dehydrogenase subunit C
TF: Transcription factor
TNI: Transcriptional network inference
VHL: von Hippel Lindau
