# Supplementary figures and images for: Master regulator analysis of paragangliomas carrying SDHx, VHL, or MAML3 genetic alterations
Source: BMC Cancer. 2019 Jun 24;19:619. doi: 10.1186/s12885-019-5813-z (PMC6591808; doi:10.1186/s12885-019-5813-z)

# SDH-VHL Overlap

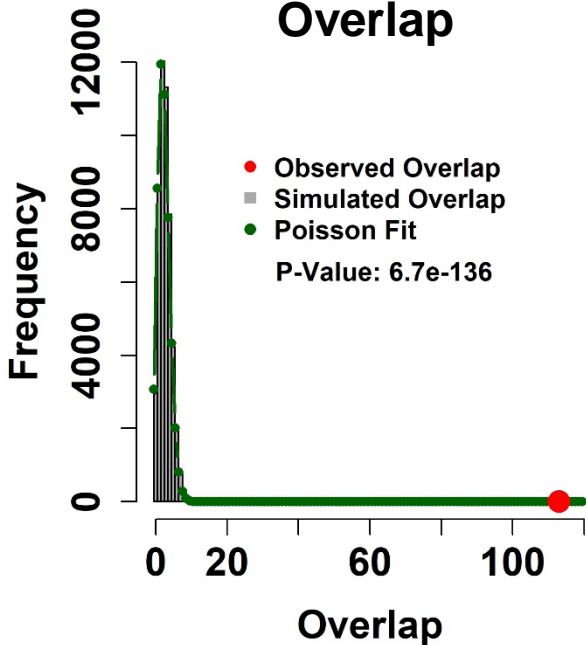

Figure S1

Supplement: Supplementary file 12 — Figure S1. Statistical analysis of differential expression gene set overlaps for SDH-loss and VHL-loss PPGL tumor molecular subtypes. Statistical simulations assessing the probability of the observed differential expression gene set overlaps. Gray histogram bars show the distribution of overlaps for randomly-selected gene sets of the same size as those analyzed. Green dots and lines show Poisson fit to the simulated data and estimated p-value for the observed overlap relative to the simulated overlap distribution. (PDF 156 kb) [file 12885_2019_5813_MOESM12_ESM.pdf]

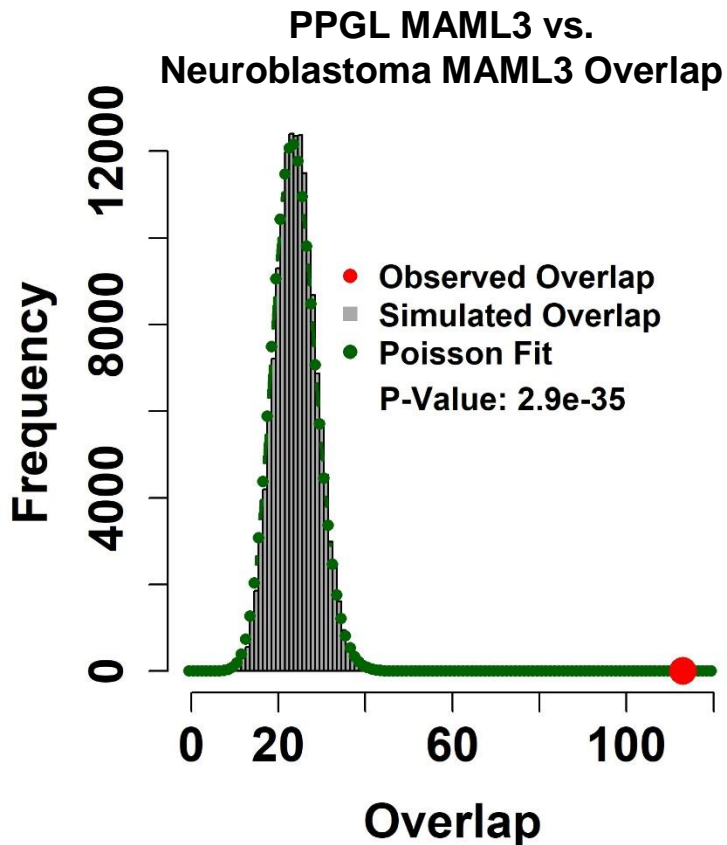

**Figure S2**

Supplement: Supplementary file 13 — Figure S2. Statistical analysis of differential expression gene set overlaps for MAML3 translocation-positive PPGL tumors and MAML3 translocation-positive neuroblastoma tumors. Statistical simulations assessing the probability of the observed differential expression gene set overlaps. Gray histogram bars show the distribution of overlaps for randomly-selected gene sets of the same size as those analyzed. Green dots and lines show Poisson fit to the simulated data and estimated p-value for the observed overlap relative to the simulated overlap distribution. (PDF 73 kb) [file 12885_2019_5813_MOESM13_ESM.pdf]

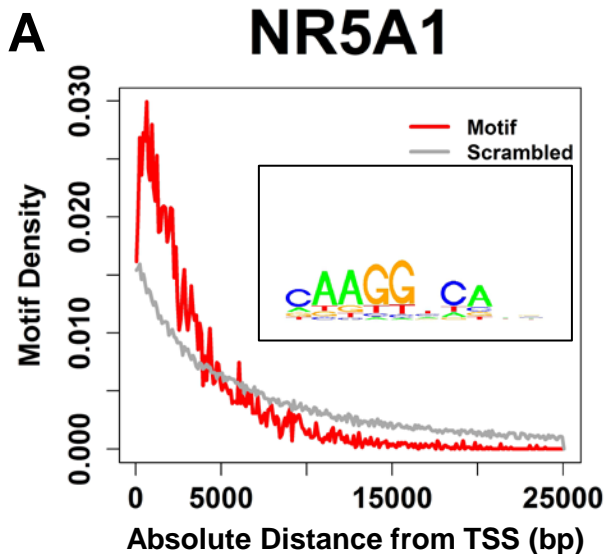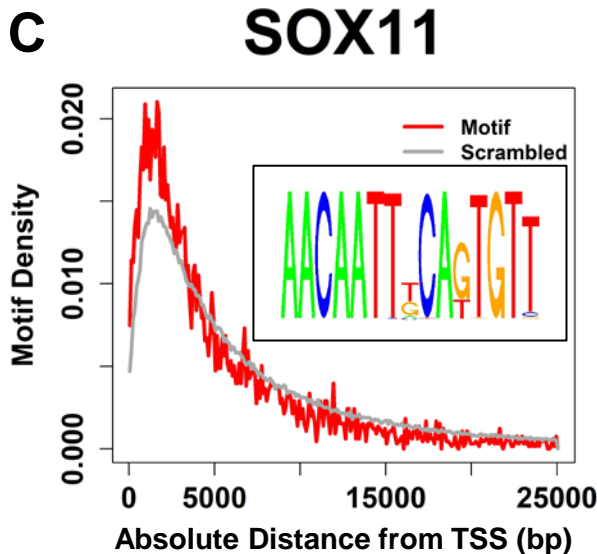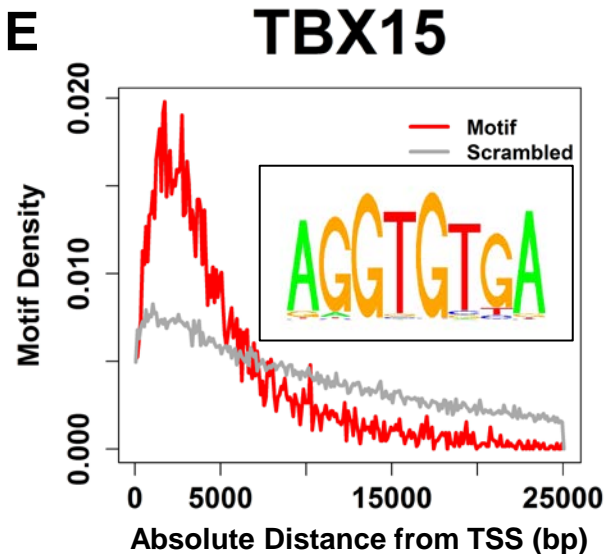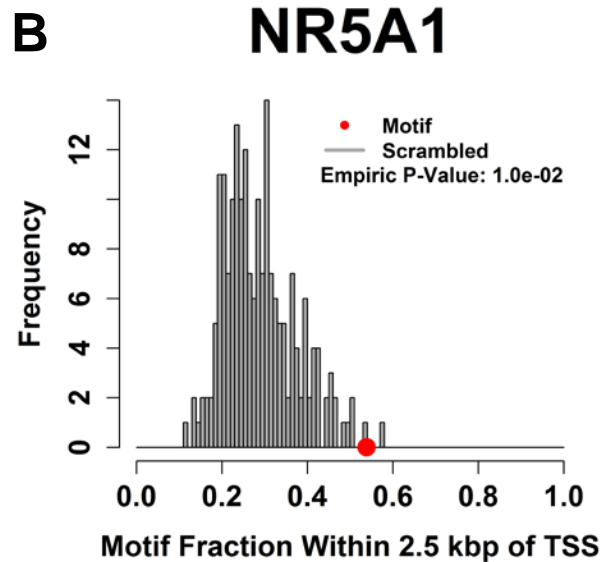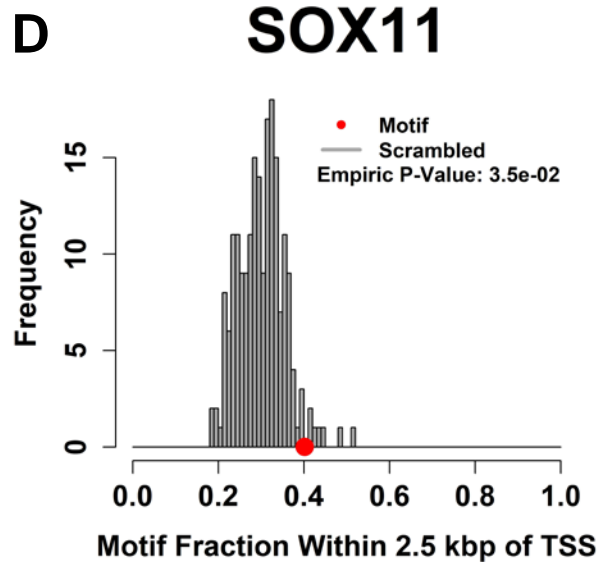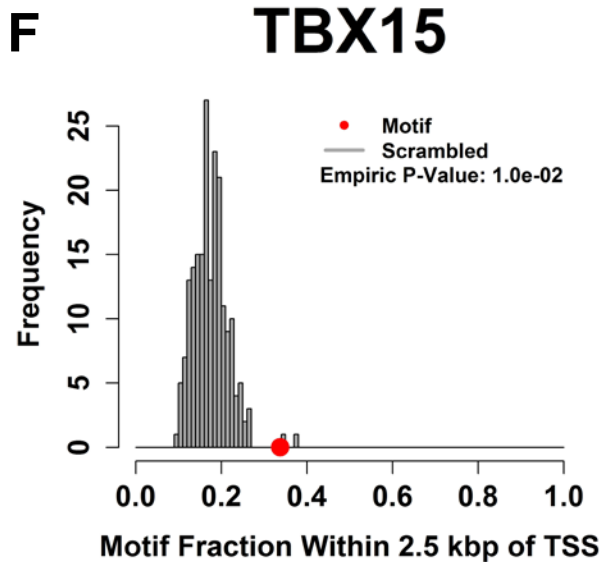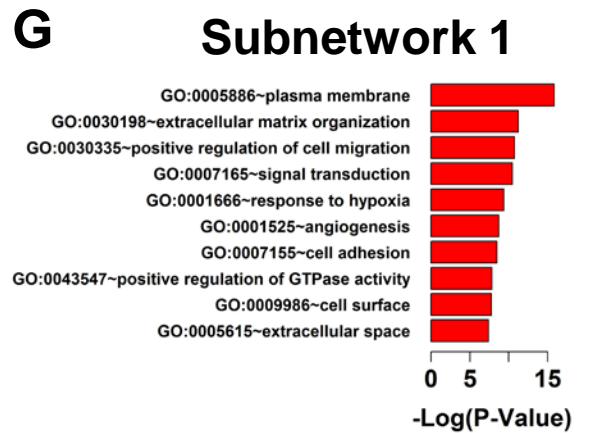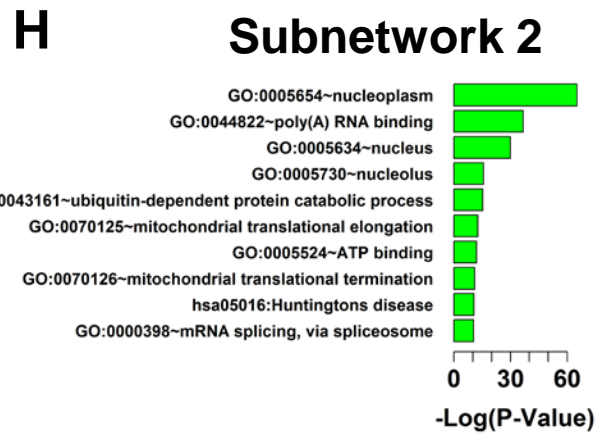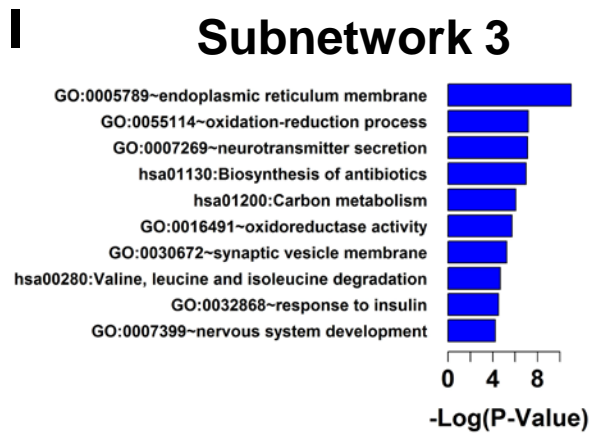

**Figure S3**

Supplement: Supplementary file 14 — Figure S3. PPGL transcriptional network validation by analysis of known TF-binding DNA motifs in inferred MR regulons and analysis of transcriptional subnetworks. A,C,E) Red traces show distribution of nearest pattern match for known TF-binding DNA motifs in inferred TF regulon. Gray traces show average nearest pattern match for scrambled version of the same motif. B,D,F) Statistical analysis of regulon motif pattern searching. Red dot indicates the fraction of nearest pattern matches for the original motif localizing to within 2.5 kbp of the TSS. Gray bars show the distribution of values yielded from the same quantification performed on scrambled versions of the original motif. Empiric p-values were estimated from the data in the random distribution and expected likelihood of the observed motif fraction with 2.5 kbp of the TSS. G-I) Analysis of transcriptional subnetwork-specific functional term enrichment among inferred target genes. Shown are the top 10 gene ontologies and/or KEGG pathways unique to each subnetwork. Subnetworks refer to those specified in Fig. 2b. (PDF 470 kb) [file 12885_2019_5813_MOESM14_ESM.pdf]

## SDH vs. VHL PPGL MR Overlap

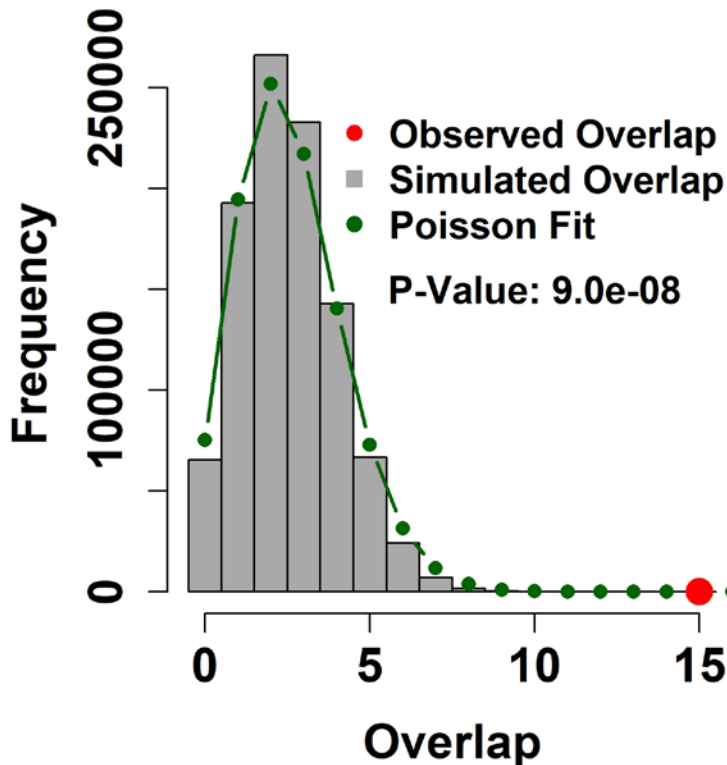

Figure S4

Supplement: Supplementary file 15 — Figure S4. Statistical analysis of master regulators inferred in SDH-loss and VHL-loss PPGL tumors. Statistical simulations assessing the probability of the observed differential expression gene set overlaps. Gray histogram bars show the distribution of overlaps for randomly-selected gene sets of the same size as those analyzed. Green dots and lines show Poisson fit to the simulated data and estimated p-value for the observed overlap relative to the simulated overlap distribution. (PDF 85 kb) [file 12885_2019_5813_MOESM15_ESM.pdf]

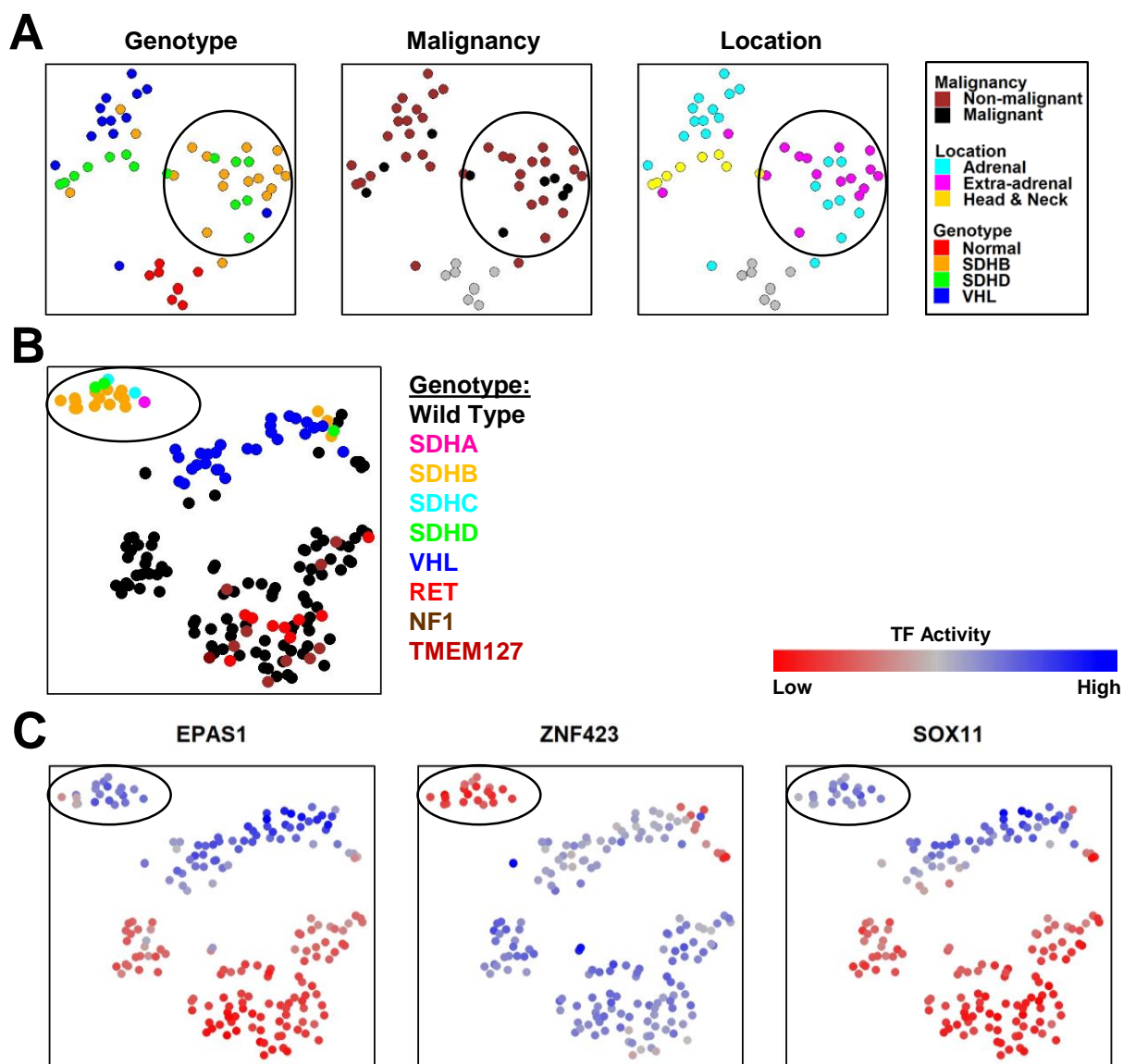

**Figure S5**

Supplement: Supplementary file 16 — Figure S5. t-SNE clustering of PPGL tumors by inferred transcription factor activity profile. A) t-SNE clustering of discovery cohort PPGL tumors by transcription factor activity profile. Colors of the data points correspond to annotations for tumor genotype, malignancy, and location, as indicated. B) t-SNE clustering of COMETE validation cohort PPGL tumors by transcription factor activity profile. Colors of the data points correspond to annotations for tumor genotype, as indicated. C) Assessment of EPAS1, ZNF423, and SOX11 activities in validation cohort specimens. Clustering pattern corresponds to genotype annotations given in panel B. (PDF 150 kb) [file 12885_2019_5813_MOESM16_ESM.pdf]
