# Supplementary material for: Master regulator analysis of paragangliomas carrying SDHx, VHL, or MAML3 genetic alterations
Source: BMC Cancer. 2019 Jun 24;19:619. doi: 10.1186/s12885-019-5813-z (PMC6591808; doi:10.1186/s12885-019-5813-z)
Supplement: Supplementary file 17 — Figure S6. Analysis of transcription factor activity profiles of SDHD-null head and neck tumors. A) Analysis of differential SDH-loss PPGL master regulator activity in SDHD-null tumors of the abdomen and thorax vs. head and neck tumors. X-axis indicates log2(fold change) in inferred transcription factor activity between tumors of the and thorax relative to head and neck tumors. Y-axis indicates degree of statistical significance for the comparison. The subset of data with adjusted p-value < 0.05 are plotted in green and include a text label. B-E) Boxplots showing distribution of activity profiles for selected differentially active SDH-loss MRs. (PDF 174 kb) [file 12885_2019_5813_MOESM17_ESM.pdf]

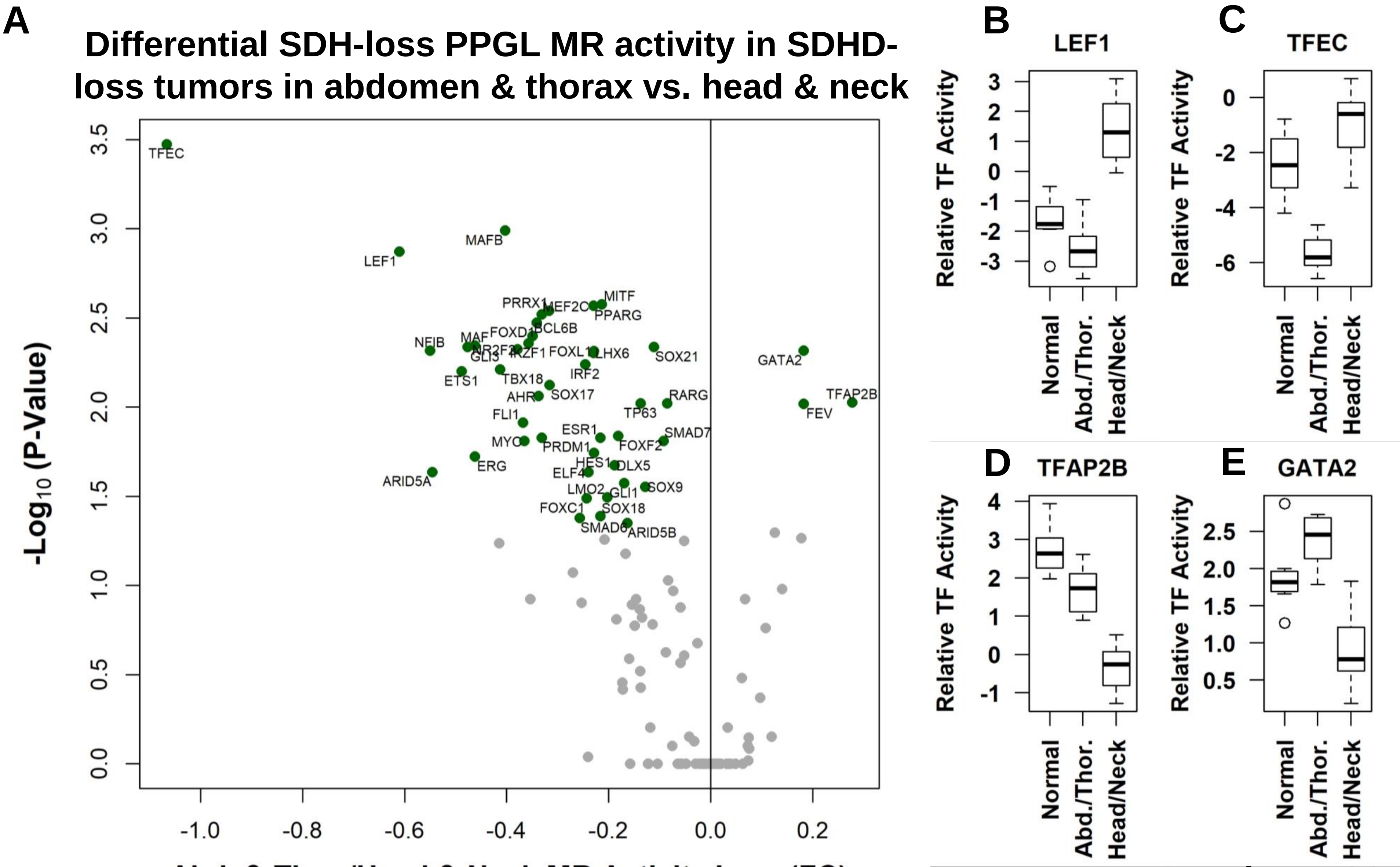
A
Differential SDH-loss PPGL MR activity in SDHD-loss tumors in abdomen & thorax vs. head & neck
-Log10 (P-Value)
Abd. & Thor./Head & Neck MR Activity Log2 (FC)
TFEC
MAFB
LEF1
PRRX1
MEF2C
MITF
PPARG
FOXD1
BCL6B
NFIB
MAF
NR2F2
GLI3
IKZF1
FOXL1
LHX6
SOX21
GATA2
ETS1
TBX18
IRF2
AHR
SOX17
RARG
TFAP2B
TP63
FEV
FLI1
ESR1
SMAD7
MYC
PRDM1
FOXF2
ERG
HES1
DLX5
ELF4
ARID5A
LMO2
GLI1
SOX9
FOXC1
SOX18
SMAD6
ARID5B
B
LEF1
C
TFEC
D
TFAP2B
E
GATA2
Relative TF Activity
Normal
Abd./Thor.
Head/Neck

Figure S6
