# Supplementary material for: Master regulator analysis of paragangliomas carrying SDHx, VHL, or MAML3 genetic alterations
Source: BMC Cancer. 2019 Jun 24;19:619. doi: 10.1186/s12885-019-5813-z (PMC6591808; doi:10.1186/s12885-019-5813-z)
Supplement: Supplementary file 18 — Figure S7. Analysis of RBP1 expression in validation cohort PPGL specimens. A-B) t-SNE clustering of COMETE validation cohort PPGL tumors by transcriptional profile. Colors in panel A indicate relative degree of RBP1 expression (red = low, blue = high). Colors in panel B correspond to annotations for tumor genotype, as indicated. (PDF 197 kb) [file 12885_2019_5813_MOESM18_ESM.pdf]

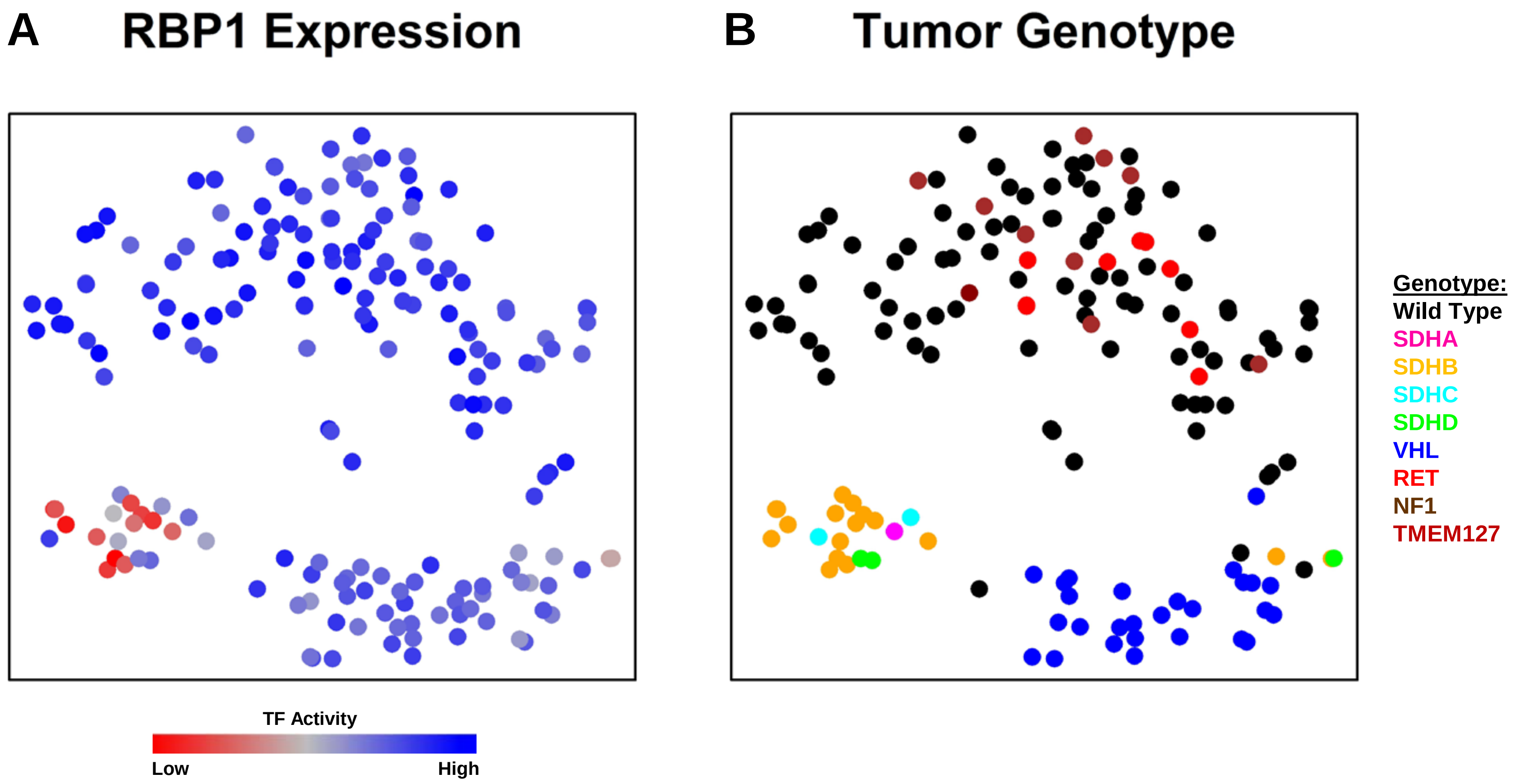
A
RBP1 Expression
TF Activity
Low
High
B
Tumor Genotype
Genotype:
Wild Type
SDHA
SDHB
SDHC
SDHD
VHL
RET
NF1
TMEM127

Figure S7
